# Binary Polymeric System Based on Fish Collagen and Poloxamer 407: Mechanical and Rheological Analysis for Pharmaceutical and Biomedical Applications

**DOI:** 10.1021/acsomega.5c04058

**Published:** 2025-11-05

**Authors:** Denise Tiemi Uchida, Douglas Shiguero Takano Ogassawara, Marcos Luciano Bruschi

**Affiliations:** Laboratory of Research and Development of Drug Delivery Systems, Department of Pharmacy, 42487 State University of Maringa, 87020-900 Maringa, Paraná, Brazil

## Abstract

Tilapia skin collagen is recognized for its regenerative properties and biocompatibility. Poloxamer 407 (P407) is a copolymer considerate ideal for stabilization and emulsification. The objective of this study was to develop a binary polymer system composed of tilapia skin collagen and P407, seeking to optimize its properties for pharmaceutical and biomedical applications. Using the two-block Box–Behnken 3^3^ factorial design, the mechanical and rheological analysis of the binary polymer systems was evaluated, varying the temperature, polymer concentration and the combination of components. The binary polymer systems demonstrated pseudoplastic behavior (shear thinning). The results indicate that the combination of tilapia collagen with P407 provides synergistic behavior, promoting desirable characteristics for pharmaceutical and biomedical applications, especially for topical use and 3D printing. The CP12 system (0.75/17.5/7%—COL/P407/Gly) stood out as the most efficient for topical application, presenting pseudoplastic behavior, significant thixotropic hysteresis area at 34 °C and viscoelastic properties. Furthermore, it exhibited adequate mechanical strength, which indicates good ability to maintain the integrity of the formulation after application. In addition, the combination of collagen and P407 offers a structural network capable of creating innovative drug delivery systems, such as 3D printing of biomedical constructs. Based on the mechanical and rheological data, the CP2, CP8, pCP2 and pCP8 systems showed pseudoplastic behavior and structural recovery capacity after shearing, essential characteristics for extrusion 3D printing. SEM analysis of the four best-performing systems revealed distinct microstructural features that correlate with mechanical and rheological properties, supporting their suitability for 3D printing. These results suggest that the hydrogels can flow through the needle and recover their original shape after deposition, which can favor the stability and fidelity of the printed structures, reinforcing their potential for biomedical applications.

## Graphical abstract

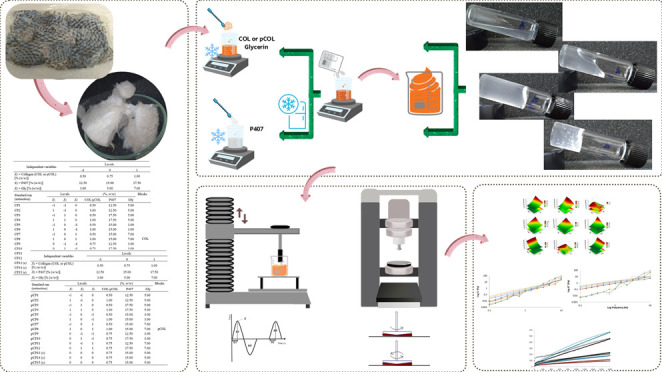

## Introduction

The search for alternative sources of collagen has intensified in recent decades, especially with the growing interest in natural and sustainable products. Collagen extracted from fish skin has emerged as a promising alternative to bovine and porcine collagen, due to its bioactive properties and lower risk of contamination by zoonotic pathogens. Furthermore, collagen derived from fish has greater cultural and religious acceptance, serving as a viable alternative for individuals who avoid porcine or bovine derived products due to ethical, religious, or dietary preferences.^1,2^ This collagen, widely used in cosmetics, food and even in the biomedical field, has advantages such as less concern with ethical and safety issues.^2–4^ Its extraction, however, requires specific techniques to ensure the preservation of its structural and functional properties. Acid extraction with acetic acid and the use of acid with pepsin are common methods that allow the efficient release of collagen, maintaining its integrity and biological potential.^5^ Extraction with acetic acid is widely used to solubilize collagen from fish skin, promoting the breakdown of cross-links between collagen fibers, which facilitates its release in soluble form. Acetic acid, being a weak acid, acts more gently on collagen structures, keeping their primary structure intact.

Extraction with pepsin, a proteolytic enzyme, is an effective method for breaking down long collagen chains into smaller fragments, which can improve their bioavailability and functionality.^6,7^ Pepsin acts specifically by removing telopeptide peptides (nonhelical ends) from collagen, regions that do not participate in the triple helix structure and that are responsible for part of the immunogenicity and spontaneous cross-linking between fibers.^8^ This modification significantly reduces the immunogenicity of collagen, making it safer for clinical applications, such as drug delivery systems, scaffolds for tissue engineering and injectable formulations. Thus, the use of pepsin in extraction represents a valuable strategy to expand the versatility and applicability of collagen in different therapeutic and technological contexts. By these two methods, it is possible to obtain collagen with good solubility properties and functionality suitable for various applications.^9,10^

Collagen extracted from tilapia skin (*Oreochromis niloticus*) is a promising alternative due to its superior mechanical and chemical properties. Tilapia is one of the most widely produced and consumed aquaculture species, which results in a large amount of waste, especially skin, which is often discarded by the fishing and fish processing industries.^11^ The use of this byproduct not only helps reduce the environmental impacts caused by the improper disposal of organic waste but also promotes sustainability by reusing materials previously considered waste. Comparatively, tilapia collagen demonstrates greater mechanical strength and thermal stability, essential characteristics for the development of durable and efficient biomaterial systems.^12^ Regarding mechanical properties, tilapia collagen also presents competitive performance. Collagen fibers maintain good structural integrity after processing, which favors its application in the production of biomembranes, three-dimensional scaffolds and controlled release systems.^13^

Compared to collagen from other tropical fish, tilapia collagen demonstrates greater thermal stability, which expands its possibilities for industrial use.^14^ This stability and resistance make the material suitable. Additionally, because it is of aquatic origin, it presents a lower risk of contamination by pathogens such as prions and viruses associated with terrestrial mammals.^15,16^ Chemically, tilapia skin has a structure less prone to microbial contamination due to its denser and stronger fibrillar network, which also contributes to a reduced risk of unwanted immune reactions, a crucial factor in biomedical applications.^17–19^ These aspects make tilapia skin collagen an advantageous alternative, not only because of its strength. Although its denaturation point is slightly lower than that of mammalian collagen, its use should not be ruled out, as studies show that fish collagen is more bioavailable and absorbs up to 1.5 times better than bovine or porcine collagen.^20^ Furthermore, its stability can be adequately controlled through modification techniques.^15^

In addition, tilapia collagen has several benefits in the pharmaceutical and biomedical fields, notably due to its regenerative, anti-inflammatory and biocompatible properties. Its ability to stimulate cell regeneration and promote wound healing makes it especially useful in the treatment of burns, ulcers and other skin lesions.^21,22^ Several studies have characterized tilapia skin collagen and demonstrated, by using techniques such as SDS-PAGE, FTIR and spectroscopy, that it is composed mainly of type I collagen, the same type predominant in mammalian skin.^16,23–26^ This type of collagen is known for its high mechanical strength and ability to promote tissue regeneration and is widely used in biomedical applications. Furthermore, tilapia collagen, because it is biocompatible and has a structure similar to human collagen, is widely accepted by the body, minimizing the risk of rejection. This collagen also has great potential for incorporation into controlled drug delivery systems, contributing to increased therapeutic efficacy, and can also be used in tissue engineering, aiding in the regeneration of cartilage, bone, and other damaged tissues.^27^ In general, collagen extracted from tilapia skin offers significant mechanical advantages compared to other collagen sources, such as bovine and porcine collagen, particularly due to its greater tensile strength and lower risk of inducing immune reactions. These properties are particularly valuable in applications such as tissue engineering and the development of controlled drug delivery systems. Furthermore, the abundance and relatively affordable cost of this collagen make it a viable and sustainable alternative. These characteristics highlight tilapia collagen as a promising option for the formulation of hydrogels, either isolated or combined with other polymers, expanding its potential application in several areas.

Previous studies have shown that concentrations of collagen extracted from tilapia skin between 0.3% and 1% are effective in biomedical formulations.^28,29^ Studies indicate its use in bone tissue engineering, associated with inorganic materials, favoring osteogenesis and offering a viable alternative to mammalian collagen.^30^ In tendon engineering, decellularized tilapia collagen matrices have proven effective as bioactive scaffolds for tissue regeneration, promoting stem cell differentiation and the formation of new tissues in in vivo models.^31^ In addition, nanofibers and sponges based on tilapia collagen have been developed as functional dressings, with properties that favor cell adhesion, skin healing and hemostasis, presenting good biocompatibility and performance comparable to or superior to commercial materials.^32,33^ Studies have also demonstrated that collagen extracted from tilapia scales can be incorporated into PVA fibrous membranes by electrospinning, forming scaffolds with good physicochemical and biological properties.^34^ These structures were biocompatible and promoted the proliferation of human fibroblasts in vitro, demonstrating their potential for applications in tissue engineering, such as graft substitutes and cartilage regeneration. Such evidence reinforces the versatility of tilapia collagen as a promising biomaterial in several therapeutic, biomedical and pharmaceuticals fields.

In addition to collagen, poloxamer 407 (P407) has emerged as a key component in the formulation of biomimetic and cosmetic systems. P407 is a triblock copolymer of polyethylene glycol (PEG) and polypropylene glycol (PPG), widely used due to its surfactant, emulsification and stabilization properties.^35,36^ The desired properties of P407 will depend on the concentration used, which can range from 5% to 30%.^37,38^ In formulations involving collagen, P407 can improve the homogeneity of the mixture, increase viscosity and help control the release of the active ingredients, such as fish collagen. Its ability to form micelles and stabilize systems with different liquid and solid phases makes it an ideal choice for creating efficient and stable polymeric systems. Although there are currently no widely published studies on the specific combination of tilapia skin collagen and P407, the combination of biocompatible polymers such as collagen with materials like P407 has great synergistic potential. This combination could optimize properties such as fluidity, mechanical stability, and controlled release, making it a promising alternative for creating bioactive hydrogels for various therapeutic and biomedical applications.

Glycerin is a viscous translucent liquid widely used in different fields (pharmaceutical, cosmetic, biodiesel or food), where it is mainly used as a humectant, plasticizer, thickener, lubricant, sweetener or antifreeze.^39–42^ With the intention of using these systems as printed biofilms, and knowing that films containing collagen are fragile, brittle and nonpeelable, in the systems developed in this manuscript, glycerin was incorporated into the formulation as a plasticizer, in order to reduce fragility and increase the flexibility and peel ability of films containing collagen. Most studies available in the literature on plasticized systems based on gelatin or collagen describe the effects of adding glycerin in low to moderate concentrations, generally ranging from 0% to 50% (w/w in relation to the polymer).^43–45^ The use of glycerin as a plasticizer has been evaluated in studies with biofilms and its results have been promising, altering the mechanical and physical properties of biofilms.^41,46^

The idea of combining fish skin collagen, P407 and glycerin in a binary polymer system is innovative, as it aims to optimize the properties of each of these components. Glycerin, in turn, acts as a humectant, maintaining skin hydration and providing the softness necessary for the formulation. P407 can act as a stabilizer and emulsifier, while fish collagen contributes with its regenerative and firming properties. By combining these ingredients, it is expected to obtain a polymer system with synergistic characteristics, which can be used in cosmetic, pharmaceutical or even biomedical applications.

However, to ensure that the polymer system has the desired effectiveness, it is essential to perform texture and rheology profile analyses of the formulations. The texture profile provides information about the physical and mechanical properties of the formulation, such as viscosity, elasticity and shear strength, which are essential for assessing the acceptance of the product and its functionality. Rheological analysis, on the other hand, allows the evaluation of the behavior of the formula under different stress and deformation conditions, which is crucial to guarantee its stability and performance in different types of applications. These analyses are essential for optimizing the system and ensuring that the final product has the desired characteristics for its applications. The aim of this study was to develop a binary polymer system with collagen obtained from tilapia skin, P407 and glycerin, aiming to optimize its properties for various applications. Texture and rheology analyses were performed to evaluate the functionality and stability of the system.

## Materials and Methods

### Samples and Chemicals

The Nile tilapia skin (*O. niloticus*) was obtained from a fish slaughterhouse, located in Umuarama city, Parana State, Brazil. Impurities such as scales, blood, meat and fat were removed, and the skin was washed with sterile 0.9% saline solution. After this process, the skins were cut into squares (5 × 5 cm), stored in plastic packaging, frozen and transported in a cooler to the Laboratory of Research and Development of Drug Release Systems (LABSLiF) of the State University of Maringa (UEM). Upon arrival at the laboratory, the material was kept in a freezer for later analysis.^47^ Glycerin (Gly), sodium hydroxide and sodium chloride (NaOH) were purchased from Synth (Sao Paulo, SP, Brazil). Acetic acid (CH_3_COOH) was obtained from Vetec (Duque de Caxias, RJ, Brazil). Poloxamer 407, pepsin from porcine gastric mucosa (≥400 units/mg protein) and cellulose acetate membrane for dialysis tubing (12,400 Da, 76 mm) were purchased from Sigma-Aldrich (Sao Paulo, SP, Brazil). Unless specified, all reagents were utilized without further purification.

### Collagen Sample

In this study, two methods of collagen extraction from tilapia skin were used: acid (COL) and acid with pepsin (pCOL). To obtain COL, the skin was previously cleaned, weighed and degreased in a 10% NaOH solution (0.1 N; pH 12) with the aid of a mechanical stirrer. The skin was washed with cold purified water until neutral. Afterward, a 20% acetic acid solution (0.5 M) was added, and agitation was maintained at 350 rpm. After extraction, the sample was centrifuged for 40 min at 2478*g* and the supernatant was collected (2×). The entire process was carried out at a temperature of 10 °C. To purify the extract, the saline precipitation method with sodium chloride (0.9 M NaCl) was performed. The extract remained refrigerated “overnight” and was centrifuged for 40 min at 2478*g*. The supernatant was discarded, and the precipitated extract was resuspended with 0.5 M acetic acid. Afterward, the sample was dialyzed in 0.1 M acetic acid for the first 24 h under refrigeration and in the following days it was dialyzed in cold purified water and kept under refrigeration. Finally, the extract was frozen and lyophilized. Moreover, another extraction was performed using pepsin and the same methodology previously described. However, an aqueous solution of acetic acid (0.5 M) containing 0.1% pepsin was added to the supernatant to remove the telopeptides^48^ and the extraction procedure was conducted more twice. Because this is a study that exclusively used biological samples of animal origin from a slaughterhouse, without any direct handling of live animals by the researchers, this study was exempted from submission to the Ethics Committee on the Use of Animals for ethical analysis.

### Preparation of Systems

The formulations of thermoresponsive binary polymer systems were prepared according to the cold method described by Schmolka.^49^ The binary systems consisted of a combination of tilapia skin collagen (COL and pCOL) and P407.

The polymer systems were designed and executed using a Box–Behnken 3^3^ design, in two blocks (COL and pCOL) totaling 15 formulations for each type of extraction (Table 1). The levels of the factors studied were *X*_1_ [COL or pCOL—0.5%; 0.75%; 1.0% (w/w)], *X*_2_ [P407—12.5%; 15.0%; 17.5% (w/w)] and *X*_3_ [Gly—3.0%; 5.0%; 7.0% (w/w)]. The response values were predicted by the quadratic polynomial equation (eq 1)­

1 $$y=\beta_{0}+\sum \beta_{ii}X_{i}+\sum \beta_{ii}X_{i}^{2}+\sum \beta_{ij}X_{i}X_{j}$$

where *Y* is the predicted value; β_0_ is the constant coefficient; β_*i*_, β_*ii*_ and β_*ij*_ are the regression coefficients of the model; and *X*_*i*_ and *X*_*j*_ represented the independent variables in the form of coded values.

**Table 1:** Box–Behnken 3^3^ Design of Formulations Containing Collagen Obtained by Acid Extraction (COL) and Acid Extraction with Pepsin (pCOL)^a^

	Levels		
Independent variables	–1	0	1
*X*_1_ = Collagen (COL or pCOL) [%, (w/w)]	0.50	0.75	1.00
*X*_2_ = P407 [% (w/w)]	12.50	15.00	17.50
*X*_3_ = Gly [% (w/w)]	3.00	5.00	7.00

	Levels			(%, w/w)			
Standard run (extraction)	*X*_1_	*X*_2_	*X*_3_	COL/pCOL	P407	Gly	Blocks
CP1	–1	–1	0	0.50	12.50	5.00	COL
CP2	1	–1	0	1.00	12.50	5.00	
CP3	–1	1	0	0.50	17.50	5.00	
CP4	1	1	0	1.00	17.50	5.00	
CP5	–1	0	–1	0.50	15.00	3.00	
CP6	1	0	–1	1.00	15.00	3.00	
CP7	–1	0	1	0.50	15.00	7.00	
CP8	1	0	1	1.00	15.00	7.00	
CP9	0	–1	–1	0.75	12.50	3.00	
CP10	0	1	–1	0.75	17.50	3.00	
CP11	0	–1	1	0.75	12.50	7.00	
CP12	0	1	1	0.75	17.50	7.00	
CP13 (c)	0	0	0	0.75	15.00	5.00	
CP14 (c)	0	0	0	0.75	15.00	5.00	
CP15 (c)	0	0	0	0.75	15.00	5.00	
pCP1	–1	–1	0	0.50	12.50	5.00	pCOL
pCP2	1	–1	0	1.00	12.50	5.00	
pCP3	–1	1	0	0.50	17.50	5.00	
pCP4	1	1	0	1.00	17.50	5.00	
pCP5	–1	0	–1	0.50	15.00	3.00	
pCP6	1	0	–1	1.00	15.00	3.00	
pCP7	–1	0	1	0.50	15.00	7.00	
pCP8	1	0	1	1.00	15.00	7.00	
pCP9	0	–1	–1	0.75	12.50	3.00	
pCP10	0	1	–1	0.75	17.50	3.00	
pCP11	0	–1	1	0.75	12.50	7.00	
pCP12	0	1	1	0.75	17.50	7.00	
pCP13 (c)	0	0	0	0.75	15.00	5.00	
pCP14 (c)	0	0	0	0.75	15.00	5.00	
pCP15 (c)	0	0	0	0.75	15.00	5.00	

a Levels for factors and their coded values of the thermoresponsive binary polymer system formulations. Acid extraction (COL); acid extraction with pepsin (pCOL); poloxamer 407 (P407); glycerin (Gly).

First, the collagen was incorporated into glycerin and half of the ultrapure water (water purification system Evoqua Water Technologies, Pittsburgh, PA, USA) used in the system, under constant mechanical stirring until hydration and complete dispersion at a temperature of 10 °C. P407 was added to the other half of the ultrapure water, leaving the preparation to rest in the refrigerator until hydration and complete dispersion of the polymer. After approximately 12 h, the P407 solution was poured into the collagen-based system under constant mechanical stirring at a temperature of 10 °C until complete incorporation of the two systems. At the end of the preparation, all formulations were centrifuged (1394*g* for 10 min), to eliminate air bubbles, and stored in the refrigerator for at least 24 h before performing the analyses.^50–53^

### Texture Profile Analysis

The TA-XTplus texture analyzer (Stable Micro Systems, Surrey, England) was used to perform the texture profile analysis (TPA) of the formulations.^51^ A quantity of 16 g of the formulation was placed in glass vials, avoiding the formation of air bubbles. In TPA mode, a polycarbonate analytical probe (10 mm in diameter) was compressed twice inside the sample, with a speed of 2 mm/s, a depth of 15 mm and a time of 15 s between the end of the first and the beginning of the second compression. At least three repetitions of the analyses were performed for each sample, at temperatures of 10, 25, and 34 °C. From the resulting graph of force versus distance and force versus time, compressibility, adhesiveness, hardness, cohesiveness and elasticity were calculated.^50–52^

### Rheology

The rheological analyses of the systems were performed using a controlled shear stress and gradient rheometer (model MARS II, Haake Thermo Fisher Scientific Inc., Newington, Germany), in flow mode, at temperatures of 10, 25, and 34 °C, with parallel cone–plate geometry of 35 mm in diameter, separated by a fixed distance of 0.052 mm.

#### Continuous Shear (Flow) Analysis

The samples were carefully applied to the bottom plate, ensuring minimal shearing of the formulation and allowing a resting time (relaxation of the introduced stress prior to analysis) of 5 min before each determination. The upward and downward flow curves were obtained with a shear rate starting from 0 s^–1^ to 1800 s^–1^, depending on each formulation. The shear rate was increased over a period of 150 s. Then, the selection of the shear rate range was determined according to the consistency of each formulation.^50,51,54^ The upward curve was evaluated using the Oswald-de-Waele equation (power law) to obtain the consistency index (*K*) and the flow behavior index (*n*) (eq 2)^50,51,54^

2 $$\sigma =K\cdot {ẏ}^{n}$$

where σ is shear stress (Pa); *K* is consistency index (Pa·s)^*n*^; *ẏ* is rate of shear (s^–1^) and *n* is flow behavior index (dimensionless).

The Casson and Herschel–Bulkley rheological models were used to determine the yield value and are respectively presented below by eqs 3 and 4 ^55^

3 $$\sigma =\sqrt[{n}]{{\left(\sigma_{y}^{n}+\left(ẏ\cdot \eta_{p}\right)\right)}^{n}}$$

4 $$\sigma =\sigma_{y}+K\cdot {ẏ}^{n}$$

where σ is shear stress (Pa); σ_y_ is yield stress (Pa); *K* is consistency index (Pa·s)^*n*^; *ẏ* is the rate of shear (s^–1^); *n* is the flow behavior index (dimensionless) and η_p_ is Casson plastic viscosity. Furthermore, the thixotropy area was calculated using the program RheoWin 4.10.0000 (Haake).

#### Oscillatory Analysis

The oscillatory analyses were performed using the rheometer in oscillatory mode. The linear viscoelastic region (LVR) was determined by increasing the oscillatory stress (torque sweep) at a fixed frequency for each sample. A stress within the LVR was selected for subsequent frequency sweep analyses. The samples were carefully applied to the bottom plate of the rheometer, ensuring minimal shearing of the formulation and allowing a resting time (relaxation of the stress introduced before analysis).^50,51^ In a frequency range of 0.1 to 10.0 Hz, at least three replicates were analyzed for each sample. The elastic modulus (*G*′), viscous or loss modulus (*G*″), dynamic viscosity (η′) and loss tangent (tan δ) were determined using the RheoWin 4.10.0000 program (Haake).

### Scanning Electron Microscopy (SEM)

The surface characteristics of the freeze-dried binary systems (CP2, CP8, pCP2 and pCP8) were analyzed using a scanning electron microscope (Shimadzu—SS550, Tokyo, Japan). Samples were deposited on stubs and coated with colloidal gold under an argon atmosphere using a sputter coater.^56^

### Statistical Analysis

For ATP, the value of the coefficient of determination (*R* ^2^) and the analysis of variance (ANOVA) results were used to validate/accept the mathematical model. Parameters with *p*-values <0.05 were considered statistically significant. The Statistica software version 8.0 was used to generate and statistically analyze the factorial design.

To detect possible correlations between the different binary systems (CP and pCP) with the APT parameters at different temperatures, Pearson’s correlation analysis estimation was used. Pearson’s correlation coefficient is often used to assess the intensity, direction of linear relationships, and correlation between two variables. Studies to correlate the extraction, structural characterization, and collagen contents were performed using Pearson’s correlation coefficient analysis.^9,57^

For continuous shear (flow), the effect of the presence of each polymer (collagen and P407), the presence of glycerin, and the variation in their concentrations were statistically evaluated on the *K*, *n*, σ, and hysteresis area indices of the formulations, using three-way ANOVA followed by Tukey’s test, with *p* < 0.05 being considered significant.

## Results and Discussion

Subsequent analyses aim to elucidate the physical, mechanical and rheological properties of thermoresponsive binary systems containing collagen extracted from TS (COL and pCOL), P407 and glycerin. Rheological and mechanical analyses of the systems were performed at three temperatures 10 °C, 25 and 34 °C with the aim of simulating different storage, handling and application conditions of the material. The temperature of 10 °C was chosen to represent refrigerated storage conditions, commonly used to preserve the stability of sensitive formulations. Room temperature (25 °C) reflects the conditions of handling in the laboratory or use in uncontrolled environments. The temperature of 34 °C was selected because it is close to the temperature of the skin surface and is therefore the most suitable for simulating the topical application environment.^58,59^

### TPA Analysis

TPA of the Box–Behnken design formulations, including both COL and pCOL, was performed at temperatures of 10, 25, and 34 °C. According to the statistical analysis (ANOVA), the quadratic polynomial equations showed the best statistical significance based on the dependent variable. The equation terms were used when they presented *p* < 0.05. The equations were significant when they presented *p* < 0.05; their respective *R* ^2^ values are presented in Tables S1–S5. These results demonstrate the reliability of the models in representing the textural properties of the different formulations and temperatures. Furthermore, the lack of fit analysis did not present a significant value (*p* > 0.05), reinforcing the adequacy of the model (Supporting Information, Tables S1–S5). TPA is a very useful analysis for evaluating the mechanical textural properties of the materials and enables them to evaluate the capacity of the formulations to develop deformations, as well as their behavior in a reversible or nonreversible manner. The texture of gels is closely related to their structural network properties, such as density, size and uniformity of distribution of the gel pores.^60^ Furthermore, it is possible to evaluate the organization and interaction between the polymers used in the binary system, and also to analyze the action of temperature and mechanical influence on the systems, simulating the physiology of the organism. The TPA results, namely hardness, compressibility, adhesiveness, elasticity and cohesiveness, of formulations are displayed in Tables S6–S8.

For pharmaceutical formulations, it is essential to know the formulation to facilitate patient adherence to treatment and facilitate the choice of packaging. Understanding this extends to aspects such as spreadability, adhesiveness and viscosity, which are closely linked to the application site (eyes, skin, mucous membranes) and the final model of this pharmaceutical form (such as structural models obtained by 3D printing). Data such as hardness and compressibility are related to the facilitated applicability and spreadability of the formulation on the skin, respectively.^61^

Furthermore, it is important to consider the adhesiveness of these systems. Their values indicate the adhesion capacity of the product. In other words, the higher the values, the more adhesive and difficult to break the formulations are, remaining in contact with the application site for a longer period of time. Cohesiveness, on the other hand, provides us with information on how the formulation structure performs after application to the skin.^62^ Low elasticity values and high cohesiveness and adhesiveness are interesting when the purpose of applying the formulation is a location where physiological movements are inevitable, which makes it difficult to retain the formulation at the application site.^52,63^

It is known that collagen undergoes denaturation and P407 undergoes the micellization process as the temperature increases. However, the interaction between them has been studied little and understood to date. By understanding this binary system, we can find an ideal system to carry different types of drugs to different types of sites of action and, furthermore, define the best applicability for the formulations presented by the factorial. The response surface graphs for each TPA parameter showed different effects, depending on the temperature and concentration of each term in the equation (Figures 1–5).

**Figure 1:**
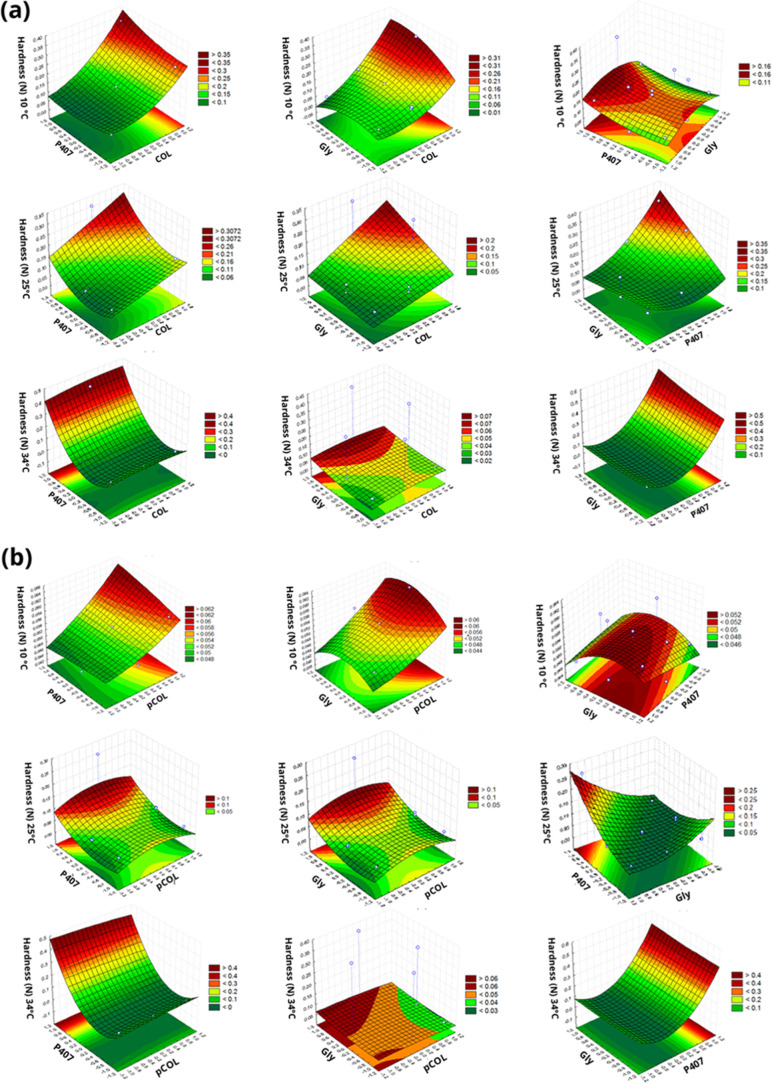
Response surface plot of the Box–Behnken factorial evaluating hardness (N) at temperatures of 10, 25, and 34 °C. (a) Acid extraction (COL), poloxamer 407 (P407) and glycerin (Gly). (b) Acid extraction with pepsin (pCOL), poloxamer 407 (P407) and glycerin (Gly).

**Figure 2:**
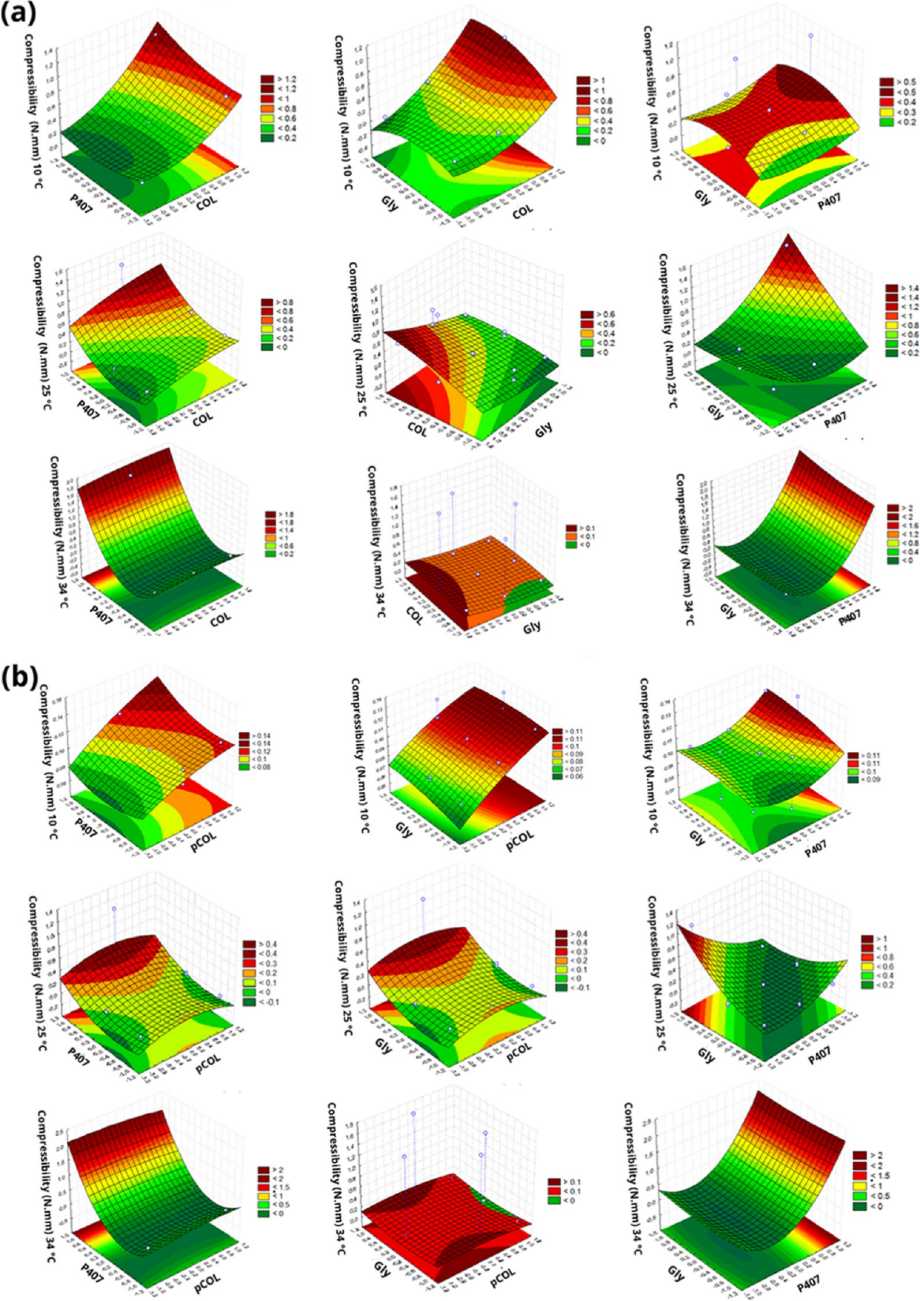
Response surface plot of the Box–Behnken factorial evaluating the compressibility (N·mm) at temperatures of 10, 25, and 34 °C. (a) Acid extraction (COL), poloxamer 407 (P407) and glycerin (Gly). (b) Acid extraction with pepsin (pCOL), poloxamer 407 (P407) and glycerin (Gly).

**Figure 3:**
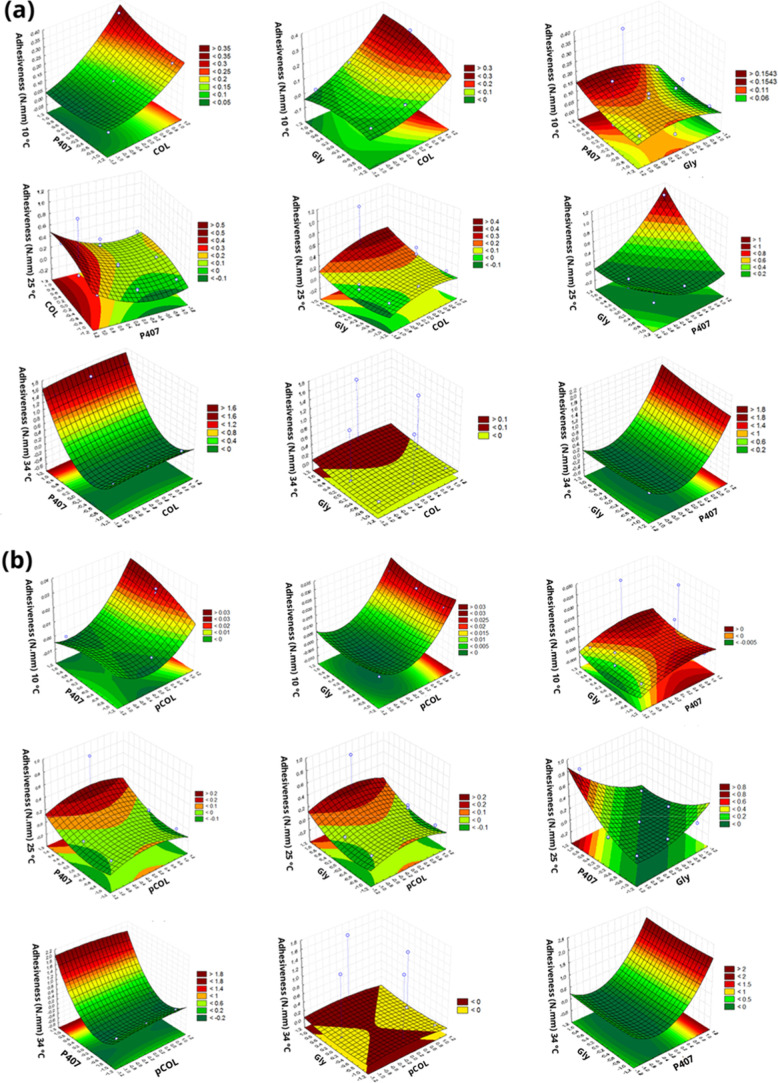
Response surface plot of the Box–Behnken factorial evaluating the adhesiveness (N·mm) at temperatures of 10, 25, and 34 °C. (a) Acid extraction (COL), poloxamer 407 (P407) and glycerin (Gly). (b) Acid extraction with pepsin (pCOL), poloxamer 407 (P407) and glycerin (Gly).

**Figure 4:**
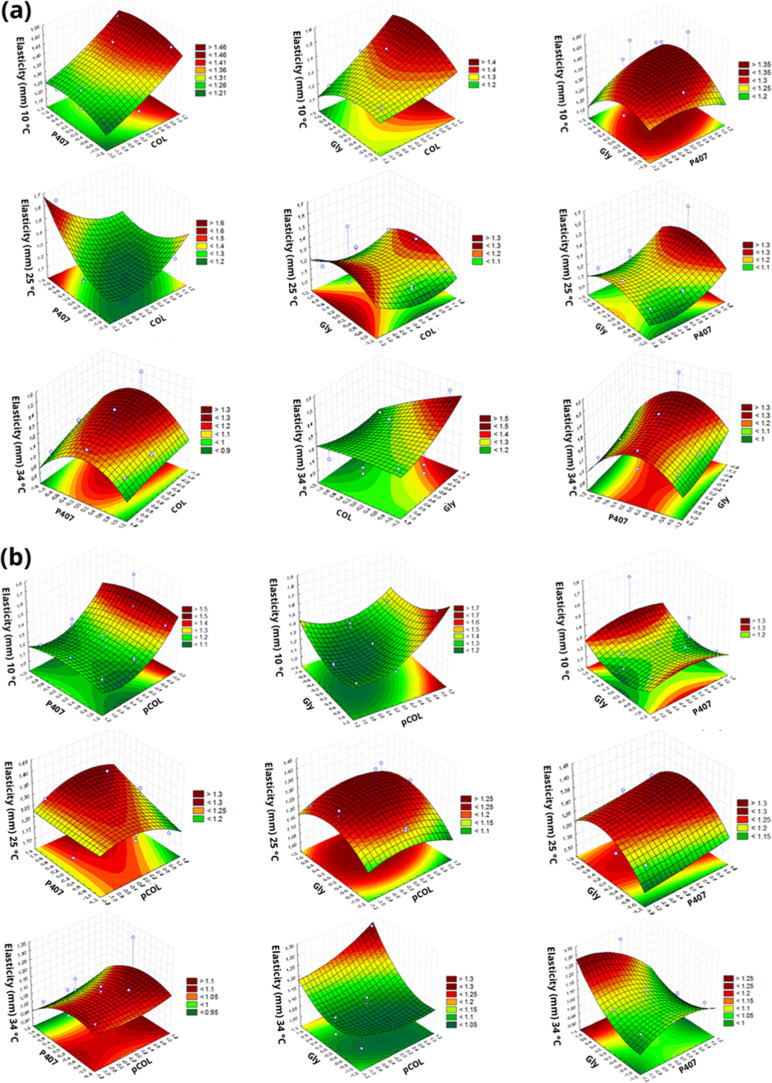
Response surface plot of the Box–Behnken factorial evaluating elasticity (mm) at temperatures of 10, 25, and 34 °C. (a) Acid extraction (COL), poloxamer 407 (P407) and glycerin (Gly). (b) Acid extraction with pepsin (pCOL), poloxamer 407 (P407) and glycerin (Gly).

Hardness is obtained by the force required to deform a sample. In other words, it can represent the stress applied to remove a pharmaceutical formulation from the packaging and apply it to the desired location (Figure 1). For the CP and pCP systems, the hardness at 10 °C was closely related to the concentration of *X*_1_. At a temperature of 25 °C, the hardness of CP was influenced by *X*_1_, *X*_2_ and *X*_3_ because it is a transition temperature for both the collagen obtained from TS and for P407. At temperature of 37 °C, the term *X*_3_ began to have a greater influence. For pCP, the same behavior was observed when comparing the hardness at different temperatures. Semisolid formulations, when they present low values of hardness and compressibility, indicate a characteristic of easy application and removal of the product.^64,65^

Compressibility is defined as the force per unit of time required to deform the sample at the time of the first compression and is illustrated in Figure 2. Its result can determine the removal of the product from the packaging material and its ability to spread at the site of action, which results in a homogeneous layer and avoiding discomfort for the patient at the time of application of the hydrogel.^53^ The compressibility for the CP and pCP systems had a greater influence of *X*_1_ at a temperature of 10 °C. In the response surface plot, for the CP systems, the compressibility was significant when the concentrations of *X*_1_ and *X*_2_ were higher at a temperature of 25 °C, while at a temperature of 34 °C the concentration of *X*_2_ had a greater influence on the compressibility than the other factors. For the pCP formulations, all factors were influential at a temperature of 25 °C; however, at a temperature of 34 °C *X*_2_ had a positive influence, unlike *X*_1_.

Adhesiveness is the value that simulates the work required to overcome the forces between the sample surface and the surface of the test that the sample came into contact with (Figure 3). It is a combination of adhesive and cohesive forces.^51–53^ For both the CP and pCP formulations, at a temperature of 10 °C, it was observed that *X*_1_ had a greater influence on the adhesiveness and as the temperature increased (25 and 34 °C) *X*_2_ began to have a greater influence.

Elasticity is used to indicate whether a hydrogel is able to return to its original position when a deformation is applied and removed. It is calculated by the ratio between the distances where the maximum force of the second compression occurs and the first compression (Figure 4). The effects of the independent factors of the CP and pCP formulations did not significantly influence the elasticity values, with the exception of the CP formulations at 25 °C.

Cohesiveness is how well a sample resists a second deformation relative to how it behaved under the first deformation. It measures how well the sample retains its structure after the first compression. High cohesion values mean high organization and performance of the product at the application site. The cohesiveness results are displayed in Figure 5. In general, all systems (CP or pCP) showed high cohesion, with values close to 1.00 at all temperatures.^51,53,66^ However, for the independent variables, the CP formulations did not present significant differences (*p* > 0.05) at any temperature. The pCP formulations at a temperature of 10 °C were influenced by *X*_2_ and at a temperature of 25 °C were influenced by *X*_3_.

**Figure 5:**
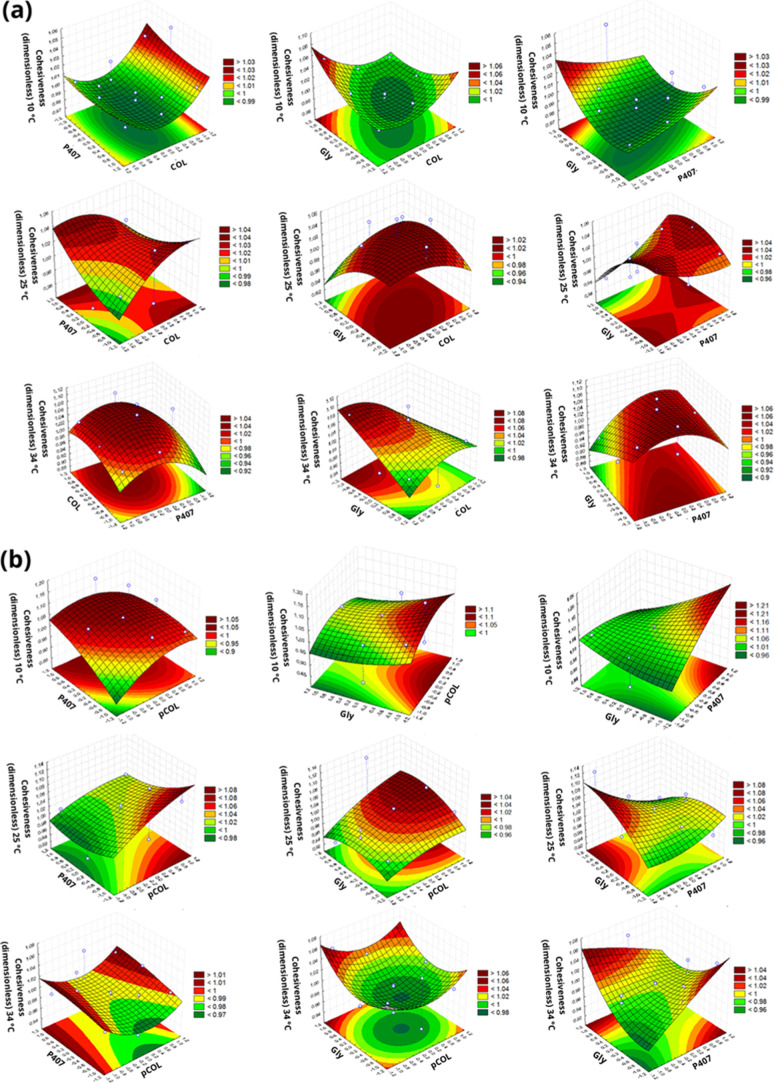
Response surface plot of the Box–Behnken factorial evaluating the cohesiveness (dimensionless) at temperatures of 10, 25, and 34 °C. (a) Acid extraction (COL), poloxamer 407 (P407) and glycerin (Gly). (b) Acid extraction with pepsin (pCOL), poloxamer 407 (P407) and glycerin (Gly).

The texture profile for all formulations showed a greater influence of *X*_1_ at a temperature of 10 °C in the highest concentrations. From a temperature of 25 °C onward, an inversion is observed and *X*_2_ begins to have a significant influence on the texture profile of the formulations. The results agree, since at low temperatures the collagen gives the formulation a greater viscosity that is lost as the temperature increases. In contrast, the presence and thermoresponsive property of the P407 copolymer gives good mechanical properties to the formulation, since at low temperatures the P407 remains in the form of monomers (fluid) and as the temperature increases micelles are formed, making the formulation more viscous. The combination of tilapia skin collagen and P407 results in a binary system with synergy and improved mechanical textural properties useful for pharmaceutical and biomedical formulations.

### Correlation of Mechanical Parameters

Studies to correlate the extraction, structural characterization and collagen contents were performed using Pearson’s correlation coefficient analysis, a *p*-value <0.05 indicates that the correlation is statistically significant (Table 2).^9,57^ The correlation coefficient for the parameter’s hardness (*R* = 0.91; 0.76; 0.99), compressibility (*R* = 0.79; 0.87; 0.99) and adhesiveness (*R* = 0.79; 0.87, 0.99) (Table 2) presented values close to 1 for all temperatures, indicating a positive correlation. As the magnitude for CP increases, that of pCP increases in the same way. The correlation among temperatures becomes greater at 34 °C, indicating that these magnitudes are improved above the gelation temperatures of the system, where the micellization process of P407 makes them more structured.

**Table 2:** Correlation Analysis (*R*) between the Binary Systems (CP and pCP) and the Texture Profile Analysis (TPA) Parameters at Temperatures of 10, 25, and 34 °C

Mechanical parameters	Temperature (°C)	*R*	*p*-value
Hardness	10	0.906	<0.0001
	25	0.759	<0.005
	34	0.985	<0.0001
Compressibility	10	0.791	<0.001
	25	0.867	<0.001
	34	0.988	<0.0001
Adhesiveness	10	0.761	<0.001
	25	0.964	<0.0001
	34	0.992	<0.0001
Cohesiveness	10	–0.549	<0.05
	25	0.275	>0.05
	34	–0.046	>0.05
Elasticity	10	–0.192	>0.05
	25	–0.072	>0.05
	34	–0.414	>0.05

When analyzing the results (Table S6), it was possible to observe that for both CP and pCP, at a temperature of 10 °C, every time the collagen concentration was higher (1%), these quantities were also higher, and this increase in values was more pronounced in formulation 4 (for both CP and pCP) when the concentration of both polymers was higher.

Comparing the two blocks (CP and pCP), it can be observed that the values of the TPA parameters for the pCP systems were, at some moments, lower than for CP. This may have occurred because pepsin has the ability to cleave peptides in the telopeptide region, making collagen more soluble.^48,67^ At a temperature of 34 °C, this difference is reduced, because as the temperature increases, the denaturation temperature of collagen occurs and P407 begins to act in the binary system. As the temperature increased, it was observed that the concentration of P407 began to influence the values of the parameters analyzed by TPA, confirming what was observed in the response surface graph. At a temperature of 25 °C, collagen began to lose influence on the parameters evaluated.

However, cohesiveness and elasticity are close to 0, indicating that there is no correlation among the amounts. This reinforces the idea that the effects of the independent factors of the CP and pCP formulations did not significantly influence the cohesiveness and elasticity values. However, the results suggest that all formulations can return to their initial position, with high structural organization and performance in the application of the hydrogel.

In recent years, collagen-based gels have been widely used in the pharmaceutical and biomedical industries as scaffolds in tissue engineering and pharmaceutical formulations (hydrogels, microneedles, and injectables).^3,20,68–70^ The requirements for the textural properties of collagen gels in different application fields are not identical. For example, collagen gels with sufficient strength and elasticity for use as scaffolds for mechanical support may not be suitable for applications requiring a soft and low/medium viscosity gel, such as drug carriers.

### Rheological Analysis

The rheological analyses of the polymeric systems from the Box–Behnken 3^3^ design were evaluated at temperatures of 10, 25, and 34 °C. The studies of the rheological properties of these polymer systems allow to better understand the possible interactions between the polymers. Furthermore, understanding these systems guides in selecting the best formulations for a given pharmaceutical and even biomedical applications. The various stresses to which the formulations are exposed during the rheological analysis also help us understand how the system behaves during manufacturing, packaging, storage and also when it is being applied to the desired location.

#### Continuous Shear (Flow) Rheometry

The consistency index (*K*) is closely related to the viscosity of a fluid with a power law at low shear. Thus, when systems present high values, it is an indication of resistance to deformation due to the entanglement of the polymer chains.

A system with good flow properties is essential to ensure ease of administration. In general, when the system is in relaxed mode, the polymer chains become entangled, giving it higher viscosity. Meanwhile, at the time of administration of the system, the application of a force increases tension, and the polymer chains tend to follow the same direction of the shear, reducing their viscosity. When decreasing and stopping the application of tension, the system tends to return to its original rheological profile, due to the relaxation of the polymer chains. For better application, formulations that present pseudoplastic flow behavior have better rheological characteristics.^52,53^

The collagens (COL and pCOL) and P407 monopolymeric systems displayed different rheological flow behaviors as the temperature and concentration were changed. At the evaluated concentrations, the collagen-based monopolymeric systems (COL and pCOL) showed shear thinning rheological behavior (Figure S1) at temperatures of 10 and 25 °C. Similar results were described by Zhang and colleagues; at a temperature of 30 °C the collagen solution showed a lower viscosity than at 17.5 °C.^71^ In the latter case, the increase in temperature changed the rheological behavior from Newtonian to non-Newtonian, due to the thermoresponsive characteristic of P407.

The binary polymer systems presented nonlinear shear stress (Pa) behavior due to the structural and conformational changes caused by the shear gradient. Thus, it was observed that the binary polymer systems containing CP and pCP presented non-Newtonian behavior (Figures S2–S6). Polymers, in general, are long-chain molecules, which unravel as the shear gradient increases, causing a decrease in viscosity. The temperature and concentration of the polymers (collagen or P407) influenced the rheological behavior of the binary systems. Mainly, when considering that collagen denatures at high temperatures (viscosity decreases), while P407, with increasing temperature, becomes more viscous. All binary systems analyzed present plastic flow behavior (shear thinning) with yield value. This result is important because plastic behavior is a desirable property for pharmaceutical formulations, especially for semisolid formulations. That is, with a higher shear rate during application, the formulation will flow immediately, favoring uniform application on the skin or mucosa. At a low shear rate, the systems present rheological properties similar to those of formulations at rest.

Analysis of the rheological parameters *K* and *n* (flow behavior index) obtained by the Ostwald-de-Waele model allows us to directly correlate the viscoelastic properties of formulations with their potential applications. High *K* values indicate higher viscosity at low shear rates, which is desirable in injectable formulations and 3D printing inks, as they provide structure and mechanical strength after application or extrusion. On the other hand, moderate *K* values favor spreadability in topical applications, allowing the system to flow properly under low application forces, promoting uniform coverage without sagging. A flow index *n*, when less than 1, characterizes pseudoplastic behavior, which is essential for systems that must flow under shear (such as during topical application, extrusion, or injection) but maintain their structure when the applied force ceases. This property is especially advantageous for inks, which require rapid structural recovery after printing, and also for topical delivery systems, where a balance between ease of application and local retention is essential. Thus, the *K* and *n* parameters prove essential for adjusting formulations according to the desired delivery method, serving as predictors of technological and functional performance.

The properties of *K* and *n* of the binary polymeric systems (CP and pCP) and monopolymeric systems (collagen and P407) were obtained (Tables 3 and 4). The changes in the concentration and temperature of the monopolymeric systems of collagen and P407 influenced the rheological characteristics of the formulations. At a temperature of 10 °C, collagen was significant (*p* < 0.05), while at a temperature of 34 °C, P407 became significant (*p* < 0.05) for *n* values. When analyzing the rheological data, it was possible to note that the collagen monopolymeric systems (COL and pCOL) in all concentrations at temperatures of 10 and 25 °C and the P407 monopolymeric systems at temperatures of 25 °C (P407 17.5%) and 34 °C (15.0 and 17.5%) displayed pseudoplastic rheological behavior with yield value, while P407 at 12.5% at a temperature of 34 °C presented pseudoplastic behavior without yield value, confirmed by the values of *n* (Table 3). However, the P407 monopolymeric systems at temperatures of 10 °C (12.5, 15.0 and 17.5%) and 25 °C (12.5 and 15.0%) showed Newtonian flow behavior. The result agrees with other studies where, at low concentrations of P407 and at low temperatures, the P407-based formulations are Newtonian fluids.^72^

**Table 3:** Effects of Collagen, Poloxamer 407 (P407) and Glycerin Concentration on the Flow Behavior Index (*n*) of Monopolymer Systems and Binary Polymer Systems at Different Temperatures (10, 25, and 34 °C)

		Components (%, w/w)			Acid extraction (COL)			Acid extraction with pepsin (pCOL)		
Formulation		Collagen	P407	Glycerin	10 °C	25 °C	34 °C	10 °C	25 °C	34 °C
Monopolymeric system		0.5			0.475 ± 0.003	0.513 ± 0.005	0.000 ± 0.000	0.601 ± 0.002	0.688 ± 0.014	0.000 ± 0.000
		0.75			0.470 ± 0.001	0.447 ± 0.003	0.000 ± 0.000	0.577 ± 0.005	0.662 ± 0.003	0.000 ± 0.000
		1			0.390 ± 0.013	0.451 ± 0.003	0.000 ± 0.000	0.598 ± 0.002	0.692 ± 0.003	0.000 ± 0.000
			12.5		1.328 ± 0.158	1.132 ± 0.015	0.027 ± 0.001	1.328 ± 0.158	1.132 ± 0.015	0.027 ± 0.001
			15		1.213 ± 0.055	1.012 ± 0.009	0.242 ± 0.005	1.213 ± 0.055	1.012 ± 0.009	0.242 ± 0.005
			17.5		1.043 ± 0.015	0.976 ± 0.018	0.183 ± 0.003	1.043 ± 0.015	0.976 ± 0.018	0.183 ± 0.003
				3	0.000 ± 0.000	0.000 ± 0.000	0.000 ± 0.000	0.000 ± 0.000	0.000 ± 0.000	0.000 ± 0.000
				5	0.000 ± 0.000	0.000 ± 0.000	0.000 ± 0.000	0.000 ± 0.000	0.000 ± 0.000	0.000 ± 0.000
				7	0.000 ± 0.000	0.000 ± 0.000	0.000 ± 0.000	0.000 ± 0.000	0.000 ± 0.000	0.000 ± 0.000
Binary polymeric system	1^a^	0.5	12.5	5	0.488 ± 0.003	0.558 ± 0.009	0.642 ± 0.023	0.515 ± 0.002	0.623 ± 0.00	0.696 ± 0.002
	2^a^	1	12.5	5	0.329 ± 0.008	0.434 ± 0.012	0.499 ± 0.020	0.354 ± 0.003	0.574 ± 0.001	0.667 ± 0.002
	3^a^	0.5	17.5	5	0.565 ± 0.002	0.728 ± 0.003	0.625 ± 0.007	0.571 ± 0.001	0.805 ± 0.003	0.282 ± 0.024
	4^a^	1	17.5	5	0.343 ± 0.007	0.659 ± 0.002	0.662 ± 0.011	0.413 ± 0.002	0.779 ± 0.015	0.247 ± 0.015
	5^a^	0.5	15	3	0.494 ± 0.003	0.626 ± 0.003	0.678 ± 0.022	0.537 ± 0.001	0.881 ± 0.003	0.902 ± 0.012
	6^a^	1	15	3	0.359 ± 0.004	0.457 ± 0.017	0.575 ± 0.003	0.384 ± 0.00	0.652 ± 0.003	0.730 ± 0.001
	7^a^	0.5	15	7	0.536 ± 0.001	0.717 ± 0.009	0.517 ± 0.023	0.546 ± 0.001	0.754 ± 0.007	0.778 ± 0.008
	8^a^	1	15	7	0.342 ± 0.005	0.528 ± 0.005	0.478 ± 0.032	0.392 ± 0.002	0.725 ± 0.007	0.748 ± 0.002
	9^a^	0.75	12.5	3	0.401 ± 0.003	0.477 ± 0.005	0.578 ± 0.006	0.419 ± 0.001	0.722 ± 0.003	0.789 ± 0.004
	10^a^	0.75	17.5	3	0.485 ± 0.003	0.664 ± 0.007	0.598 ± 0.011	0.472 ± 0.005	0.892 ± 0.012	0.243 ± 0.005
	11^a^	0.75	12.5	7	0.439 ± 0.041	0.556 ± 0.01	0.702 ± 0.004	0.432 ± 0.001	0.562 ± 0.003	0.620 ± 0.001
	12^a^	0.75	17.5	7	0.482 ± 0.003	0.480 ± 0.011	0.114 ± 0.018	0.486 ± 0.002	0.637 ± 0.029	0.191 ± 0.002
	13^a^	0.75	15	5	0.441 ± 0.008	0.521 ± 0.006	0.643 ± 0.015	0.451 ± 0.000	0.796 ± 0.042	0.774 ± 0.002
	14^a^	0.75	15	5	0.431 ± 0.008	0.608 ± 0.005	0.693 ± 0.007	0.455 ± 0.002	0.823 ± 0.003	0.851 ± 0.0014
	15^a^	0.75	15	5	0.427 ± 0.004	0.597 ± 0.022	0.667 ± 0.022	0.461 ± 0.001	0.904 ± 0.005	0.897 ± 0.013

a Fifteen formulations were prepared using the Box–Behnken 3^3^ design. When acid-extracted collagen (COL) was used, the formulation is called CP. When acid–pepsin-extracted collagen (pCOL) was used, the formulation is called pCP.

**Table 4:** Effects of Collagen, Poloxamer 407 (P407) and Glycerin Concentration on the Consistency Index (*K*) of Monopolymeric Systems and Binary Polymeric Systems at Different Temperatures (10, 25, and 34 °C)

		Components (%, w/w)			Acid extraction (COL)			Acid extraction with pepsin (pCOL)		
Formulation		Collagen	P407	Glycerin	10 °C	25 °C	34 °C	10 °C	25 °C	34 °C
Monopolymeric system		0.5			0.869 ± 0.025	0.574 ± 0.023	0.000 ± 0.000	0.325 ± 0.002	0.157 ± 0.014	0.000 ± 0.000
		0.75			0.882 ± 0.012	1.378 ± 0.04	0.000 ± 0.000	0.371 ± 0.032	0.218 ± 0.002	0.000 ± 0.000
		1			3.002 ± 0.391	1.578 ± 0.075	0.000 ± 0.000	0.376 ± 0.005	0.167 ± 0.004	0.000 ± 0.000
			12.5		0.001 ± 0.000	0.005 ± 0.001	1.011 ± 0.006	0.001 ± 0.000	0.005 ± 0.001	1.011 ± 0.006
			15		0.002 ± 0.000	0.039 ± 0.002	37.347 ± 2.215	0.002 ± 0.000	0.039 ± 0.002	37.347 ± 2.215
			17.5		0.018 ± 0.002	0.155 ± 0.019	107.933 ± 4.197	0.018 ± 0.002	0.155 ± 0.019	107.933 ± 4.197
				3	0.000 ± 0.000	0.000 ± 0.000	0.000 ± 0.000	0.000 ± 0.000	0.000 ± 0.000	0.000 ± 0.000
				5	0.000 ± 0.000	0.000 ± 0.000	0.000 ± 0.000	0.000 ± 0.000	0.000 ± 0.000	0.000 ± 0.000
				7	0.000 ± 0.000	0.000 ± 0.000	0.000 ± 0.000	0.000 ± 0.000	0.000 ± 0.000	0.000 ± 0.000
Binary polymeric system	1^a^	0.5	12.5	5	2.333 ± 0.114	2.650 ± 0.078	1.806 ± 0.024	1.5 ± 0.197	0.955 ± 0.02	0.596 ± 0.013
	2^a^	1	12.5	5	22.337 ± 2.263	14.737 ± 0.716	4.932 ± 0.880	10.297 ± 0.110	1.804 ± 0.039	0.789 ± 0.048
	3^a^	0.5	17.5	5	1.625 ± 0.088	2.410 ± 0.083	4.082 ± 0.253	1.443 ± 0.141	0.977 ± 0.045	58.873 ± 7.26
	4^a^	1	17.5	5	28.193 ± 1.065	3.071 ± 0.062	3.647 ± 0.338	8.806 ± 0.814	1.421 ± 0.192	77.443 ± 9.876
	5^a^	0.5	15	3	3.890 ± 0.160	2.467 ± 0.020	2.458 ± 0.456	1.509 ± 0.124	0.179 ± 0.002	0.178 ± 0.024
	6^a^	1	15	3	13.433 ± 0.692	8.528 ± 0.328	9.812 ± 0.151	8.434 ± 1.030	1.726 ± 0.039	1.151 ± 0.168
	7^a^	0.5	15	7	2.059 ± 0.162	7.03 ± 0.123	10.063 ± 0.912	1.534 ± 0.022	0.882 ± 0.053	0.856 ± 0.072
	8^a^	1	15	7	24.647 ± 1.773	12.563 ± 0.282	16.723 ± 0.825	9.038 ± 0.166	1.346 ± 0.079	1.096 ± 0.060
	9^a^	0.75	12.5	3	8.335 ± 0.177	6.726 ± 0.344	3.746 ± 0.115	5.014 ± 0.343	0.334 ± 0.014	0.265 ± 0.009
	10^a^	0.75	17.5	3	6.236 ± 0.315	4.517 ± 0.452	6.148 ± 0.623	3.465 ± 0.411	0.389 ± 0.389	59.890 ± 3.904
	11^a^	0.75	12.5	7	7.742 ± 0.994	5.596 ± 0.177	1.265 ± 0.091	3.188 ± 0.133	2.341 ± 0.108	1.505 ± 0.049
	12^a^	0.75	17.5	7	6.944 ± 0.789	28.157 ± 1.950	251.570 ± 14.719	3.012 ± 0.140	5.598 ± 0.837	129.13 ± 0.902
	13^a^	0.75	15	5	6.3710 ± 0.340	4.949 ± 0.269	4.350 ± 0.728	5.265 ± 0.073	0.455 ± 0.055	0.609 ± 0.078
	14^a^	0.75	15	5	8.827 ± 0.570	5.345 ± 0.176	2.77 ± 0.237	3.904 ± 0.582	0.390 ± 0.02	0.310 ± 0.015
	15^a^	0.75	15	5	8.4213 ± 0.366	5.577 ± 0.595	3.810 ± 0.162	3.906 ± 0.073	0.205 ± 0.01	0.216 ± 0.018

a Fifteen formulations were prepared using the Box–Behnken 3^3^ design. When acid-extracted collagen (COL) was used, the formulation is called CP. When acid–pepsin-extracted collagen (pCOL) was used, the formulation is called pCP.

This change in P407 flow behavior with temperature change is related to its thermoresponsive properties, where the micellization process begins to occur. In situ thermosensitive systems, such as those based on P407, present a highly temperature-dependent sol–gel transition, which is directly related to the formation and organization of micelles. At temperatures below the lower critical solution temperature (LCST), these formulations remain in the solution form, with liquid behavior and low viscosity.^73^ This state is maintained due to the hydration of the polymer chains, especially of the hydrophilic poly­(ethylene oxide) (PEO) blocks, which prevent the self-association of the chains. However, as the temperature approaches or exceeds the LCST, the entropy–enthalpy balance becomes unfavorable for solubility, leading to the progressive dehydration of the hydrophobic poly­(propylene oxide) (PPO) blocks.^36,53,73^ This triggers the self-aggregation of these hydrophobic regions, which form the core of core–shell micelles, with the PEO blocks oriented toward the periphery in contact with the aqueous medium. When the concentration and temperature are sufficiently high (above the critical micellar temperature), these micelles organize themselves into compact and ordered arrangements, promoting the formation of a three-dimensional network responsible for the sol–gel transition (gelation temperature, *T*_sol/gel_). In this state, the material acquires viscoelastic behavior (more information on oscillatory rheology), with a predominance of the storage modulus (*G*′) over the loss modulus (*G*″), reflecting solid properties. The formation of the micellar network not only increases viscosity but also gives the system a characteristic pseudoplastic behavior (shear thinning): at rest, the gel maintains its structure due to the interaction between micelles; under shear stress, these interactions are temporarily undone, resulting in a decrease in viscosity and facilitating flow. This property is desirable in biomedical and pharmaceutical applications, such as sustained-release systems, as it allows administration as a solution at room temperature (facilitating application), followed by gelation at the target site (since body temperature ranges from 33 to 37 °C), promoting prolonged retention and increased bioavailability.^52,61,73–75^ Thus, thermoreversible behavior, guided by micellar interactions and their structural organization, is essential to ensure functionality, mechanical performance, and clinical viability of P407-based formulations.

It is noted that for collagen, as the temperature increased, the viscosity decreased, probably due to the denaturation of the collagen protein. This characteristic was found in another study, indicating that the increase in the interactions of collagen macromolecules leads to an increase in chain entanglement and more pronounced non-Newtonian behavior.^76^ The shear-thinning behavior of collagen can be explained in terms of the aggregation behavior of collagen molecules in solution. Since studies show that the critical concentration for collagen aggregation is 0.30 to 0.50 mg/mL, a collagen solution with a concentration of 10 mg/mL represents a stabilized aggregate state.^77^ At rest, in the absence of shearing, the collagen molecules bind together, forming a triple helix structure that exhibits an irregular order and serves to reinforce the inter- and intra-molecular frictional forces. With the application of shear stress, the force binding the collagen molecules together is gradually reduced and the collagen molecules begin to slide more easily over each other, resulting in a large decrease in internal friction. Gly alone did not have a significant *n* value at any of the temperatures.

The binary polymer systems, both for CP and pCP, showed pseudoplastic behavior (shear thinning). At a temperature of 10 °C, collagen had a significant influence (*p* < 0.05) on *n*. As the temperature increased, the inversion of the significance was visible, which became P407. Looking closely, the binary polymer systems composed of 1% collagen COL or pCOL presented more significant values. However, there was a difference between the two types of collagen extraction at a temperature of 10 °C. For COL, there was no significant difference between the monopolymeric formulation of COL at 1% and the binary polymer systems containing 1% collagen, while for pCOL, there was a significant difference between the monopolymeric and binary systems at 1% pCOL. This result suggests that for pCOL, P407 and glycerin influence the value of *n*, probably due to the lack of telopeptides removed from the collagen triple helix chain by the enzymatic action of pepsin, which result in structural modifications that affect the viscosity and elasticity of the formulation.^78,79^ At temperature of 34 °C, the concentration of P407 begins to have a significant impact (*p* < 0.05) on *n*. The highest concentration of P407 (17%), in both the monopolymeric and binary systems, had a greater influence on *n*, indicating that the presence or absence of collagen and glycerin did not significantly affect the systems.

Observing the *n* values of the monopolymeric systems, it is noted that both collagen and P407 alone did not show pseudoplastic behavior under certain conditions. The monopolymeric collagen systems did not present measurable viscosity at 34 °C, while those with only P407 showed almost Newtonian behavior at lower temperatures (10–15 °C), with discrete variations in apparent viscosity, a pattern that suggests the absence of relevant shear thinning under these conditions. However, when analyzing the binary systems containing collagen and P407 at all temperatures evaluated (10, 25, and 34 °C), a clear decrease in *n* is observed with increasing temperature, indicative of pseudoplastic behavior (shear thinning). This effect is systematically present in the binary systems, in contrast to the monopolymeric ones. This change in rheological profile suggests that the combination of the two polymers promotes physical or structural interactions that result in a supramolecular reorganization under shear. The presence of collagen can intensify this response by providing additional intertwining between the polymer chains and establishing noncovalent interactions, such as hydrogen bonds, with the P407 chains.^37,38,80–82^ These interactions favor the formation of a more cohesive polymer network that is responsive to mechanical stress, contributing to the maintenance of the system’s viscoelastic properties. This synergy between collagen and P407 is essential for obtaining formulations with adjustable rheological performance, being especially beneficial for pharmaceutical and biomedical applications, such as injectable gels, which require low viscosity during application and rapid structural recovery at the injection site, and inks for 3D extrusion printing, which require pseudoplasticity to facilitate flow under pressure and, simultaneously, the ability to maintain their shape after deposition.

*K* is related to the viscosity of a fluid with a power law at low shear (Table 4). The collagen monopolymer formulations (COL and pCOL) were characterized by a decrease in *K* as the temperature increased, due to the progressive orientation and untangling of the chains at a temperature of 10 °C. The *K* value increased when the collagen concentration was increased, and this increase was significant (*p* < 0.05) in the COL monopolymer systems. When the temperature increased, consequently there was a decrease in *K*, due to protein denaturation. The presence of the enzyme pepsin in the collagen extraction interfered with the *K* values, since the presence of the enzyme removes the telopeptides from the collagen, affecting the viscosity.^79^ The monopolymeric formulations with P407 presented the same characteristic, but more pronounced at a temperature of 34 °C. Furthermore, *K* was significant (*p* < 0.05) as the P407 concentration increased.

In binary polymer systems at a temperature of 10 °C, the *K* value was significantly different (*p* < 0.05) at the highest collagen concentration (COL and pCOL), with little influence on the concentration of P407 or Gly. It is noted that there is a difference in *K* values when comparing binary polymer systems containing COL and pCOL. Systems containing COL have higher *K* values than in the presence of pCOL. Studies show that collagen without removing telopeptides has a faster polymerization capacity, forming more consistent and resistant gels.^79^

As the temperature increased, the *K* values in the formulations with 1% collagen decreased (for both COL and pCOL), with the exception of the formulation containing 1% pCOL; 17.5% P407 and 5% glycerin. This reduction in the *K* value was visible even in binary systems containing the highest concentration of P407. Fu and collaborators found that the solution/gel transition temperature of P407 increased as the collagen concentration increased. This behavior can be attributed to the fact that this interaction between the two polymers weakens the hydrophobic interactions between the P407 chains, responsible for thermal gelation.^83^ This effect (delayed thermal gelation) ends up being beneficial, favoring the application of the binary polymer system in a minimally invasive procedure, such as the application of an injection at room temperature.

At 25 °C, the collagen concentration loses its influence, opening space for the higher concentration of P407 and Gly to exert significant effect (*p* < 0.05) on the *K* value. However, it was at 34 °C that this difference was striking. Formulations with a low collagen concentration (0.5 and 0.75%), for both COL and pCOL, because they do not have enough strength to weaken the hydrophobic interactions of P407, allow the micellization process to occur at a temperature close to 34 °C. The increase in the *K* value as the temperature was increased had a significant influence (*p* < 0.05), but formulations containing 0.5/17.5/5; 0.5/15/7; 0.75/17.5/7% (CP3; 7; 12—COL/P407/Gly) and 0.5/17.5/5; 1/17.5/5; 0.5/15/7; 0.75/17.5/7% (pCP3; 4; 7; 12—pCOL/P407/Gly). The *K* value of the binary polymeric system was higher, even higher than the monopolymeric system, at the concentration of 0.75/17.5/7%, for both the formulation containing COL and pCOL. It can be noted that for the binary systems, at a temperature of 34 °C, Gly together with P407 played an important role in the *K* value in the formulations. In studies carried out by Miller and Drabik, in a formulation composed of 25% P407 and 10% glycerin, it was observed the system increased the viscosity as the temperature increased from 5 to 15 °C; however, at low concentrations, Gly could not change the viscosity of the P407 formulation.^36^ Furthermore, Gly can affect the solution/gel transition temperature and increase the elasticity of formulations as its concentration increases.^84^

It is known that Gly can increase the viscosity of systems composed of polymers such as sodium carboxymethyl cellulose and sodium alginate. This characteristic may be associated with an improved interaction of the polymer in solution or with a specific association between the polymer and the polyhydroxy compound.^85^ In another study carried out with Carbopol 940, the authors found that Gly influenced the viscosity of the neutralized gel, probably attributing this behavior to changes in solvation and/or molecular shape, and that consistencies depend more on the nature of the solvent than on its molecular size and inherent viscosity.^86^ Another factor that must be taken into consideration is that a 0.9% NaCl solution was used for collagen precipitation. Studies show that chloride and hydrochloride ions somehow affect the resistance of a P407-based formulation to increased temperature.^84^

Based on the results obtained, it is observed that the binary systems presented significantly higher *K* values in the formulations with higher concentrations of P407 and Gly, especially at 34 °C, indicating higher viscosity at low shear rates. This behavior favors injectable applications and 3D printing, in which the material must maintain its shape and structural stability after application or extrusion. At the same time, *n* values less than 1, characteristic of pseudoplastic behavior, were observed in all binary formulations. This reinforces that the CP and pCP systems respond adequately to shear: they flow during application (injection or spreading) and recover their structure when the force is released, which is essential for maintaining the product at the application site. Therefore, the rheological parameters *K* and *n* confirm that the developed systems have adjustable and desirable properties for topical and injectable use, with potential to be explored in several pharmaceutical and biomedical applications.

The formulations were evaluated for yield values using the Casson and Herschel–Bulkley rheological models, and the hysteresis area are displayed in Tables 5 and 6, respectively. The Casson rheological model was adopted for monopolymeric systems and binary polymer systems. Systems that have yield values indicate that the material has the capacity to withstand shear stresses without starting to flow. As soon as the shear stress values exceed the yield values (σ_y_), the material starts to flow due to the weakening of its structure. Systems that have this phenomenon have been considered desirable, as it allows the molecules to be trapped in a dispersed manner in polymeric materials.

**Table 5:** Effects of Collagen, Poloxamer 407 (P407) and Glycerin Concentration on the Yield Value (σ_y_; Pa) of Monopolymeric Systems and Binary Polymeric Systems at Different Temperatures (10, 25, and 34 °C)

		Components (%, w/w)			Acid extraction (COL)			Acid extraction with pepsin (pCOL)		
Formulation		Collagen	P407	Glycerin	10 °C	25 °C	34 °C	10 °C	25 °C	34 °C
Monopolymeric system		0.5			4.782 ± 0.145	3.417 ± 0.076	0.000 ± 0.000	1.964 ± 0.028	0.906 ± 0.182	0.000 ± 0.000
		0.75			4.809 ± 0.072	6.937 ± 0.151	0.000 ± 0.000	2.170 ± 0.211	1.298 ± 0.007	0.000 ± 0.000
		1			12.437 ± 1.042	7.994 ± 0.374	0.000 ± 0.000	2.379 ± 0.020	0.876 ± 0.032	0.000 ± 0.000
			12.5		0.000 ± 0.000	0.000 ± 0.000	0.000 ± 0.000	0.000 ± 0.000	0.000 ± 0.000	0.000 ± 0.000
			15		0.000 ± 0.000	0.000 ± 0.000	91.477 ± 2.248	0.000 ± 0.000	0.000 ± 0.000	91.477 ± 2.248
			17.5		0.000 ± 0.000	0.758 ± 0.051	214.000 ± 4.454	0.000 ± 0.000	0.758 ± 0.051	214.000 ± 4.454
				3	0.000 ± 0.000	0.000 ± 0.000	0.000 ± 0.000	0.000 ± 0.000	0.000 ± 0.000	0.000 ± 0.000
				5	0.000 ± 0.000	0.000 ± 0.000	0.000 ± 0.000	0.000 ± 0.000	0.000 ± 0.000	0.000 ± 0.000
				7	0.000 ± 0.000	0.000 ± 0.000	0.000 ± 0.000	0.000 ± 0.000	0.000 ± 0.000	0.000 ± 0.000
Binary polymeric system	1^a^	0.5	12.5	5	13.757 ± 0.595	18.683 ± 0.854	15.140 ± 0.528	9.290 ± 1.289	7.457 ± 0.139	5.047 ± 0.109
	2^a^	1	12.5	5	77.280 ± 5.764	73.330 ± 1.440	29.537 ± 4.010	38.117 ± 0.771	12.913 ± 0.283	6.524 ± 0.379
	3^a^	0.5	17.5	5	11.753 ± 0.691	21.323 ± 0.830	36.843 ± 1.560	10.404 ± 1.005	7.476 ± 0.396	163.567 ± 8.486
	4^a^	1	17.5	5	105.133 ± 2.250	26.463 ± 0.513	31.283 ± 2.819	40.488 ± 4.370	11.657 ± 2.060	191.133 ± 14.066
	5^a^	0.5	15	3	23.427 ± 2.117	20.277 ± 0.085	22.000 ± 3.106	9.960 ± 0.724	0.898 ± 0.018	0.639 ± 0.106
	6^a^	1	15	3	51.140 ± 2.823	45.040 ± 1.879	72.593 ± 0.372	34.447 ± 4.142	14.393 ± 0.261	12.420 ± 1.957
	7^a^	0.5	15	7	9.651 ± 0.638	15.493 ± 0.944	70.467 ± 6.808	10.417 ± 0.114	7.605 ± 0.504	7.046 ± 0.681
	8^a^	1	15	7	87.357 ± 5.092	82.643 ± 0.531	113.133 ± 16.182	38.297 ± 0.429	11.757 ± 0.616	9.266 ± 0.5132
	9^a^	0.75	12.5	3	30.793 ± 1.055	37.960 ± 1.779	27.663 ± 0.444	23.083 ± 1.497	2.813 ± 0.104	2.071 ± 0.085
	10^a^	0.75	17.5	3	36.040 ± 1.959	35.453 ± 3.652	51.403 ± 3.922	18.913 ± 2.424	1.847 ± 0.170	145.533 ± 7.525
	11^a^	0.75	12.5	7	32.870 ± 0.550	39.337 ± 1.028	11.293 ± 0.834	15.507 ± 0.750	16.300 ± 0.783	11.813 ± 0.312
	12^a^	0.75	17.5	7	39.723 ± 4.507	165.733 ± 8.240	374.767 ± 1.721	17.353 ± 0.920	51.693 ± 10.983	257.333 ± 3.102
	13^a^	0.75	15	5	31.950 ± 1.062	31.787 ± 1.135	36.887 ± 5.808	27.137 ± 0.672	3.492 ± 0.644	5.018 ± 0.631
	14^a^	0.75	15	5	42.683 ± 1.801	42.110 ± 1.082	24.667 ± 2.211	18.533 ± 0.543	2.850 ± 0.182	1.919 ± 0.0781
	15^a^	0.75	15	5	41.177 ± 2.024	39.553 ± 1.424	30.293 ± 5.716	20.632 ± 0.328	0.785 ± 0.093	0.843 ± 0.142

a Fifteen formulations were prepared using the Box–Behnken 3^3^ design. When acid-extracted collagen (COL) was used, the formulation is called CP. When acid–pepsin-extracted collagen (pCOL) was used, the formulation is called pCP.

**Table 6:** Effects of Collagen, Poloxamer 407 (P407) and Glycerin Concentration on the Hysteresis Area (Pa/s) of Monopolymeric Systems and Binary Polymeric Systems at Different Temperatures (10, 25, and 34 °C)

		Components (%, w/w)			Acid extraction (COL)			Acid extraction with pepsin (pCOL)		
Formulation		Collagen	P407	Glycerin	10 °C	25 °C	34 °C	10 °C	25 °C	34 °C
Monopolymeric system		0.5			–266.833 ± 24.742	–789.667 ± 75.179	34,856.667 ± 3896.721	238.000 ± 44.412	–159.533 ± 27.432	1,116,666.667 ± 96,691.951
		0.75			–83.750 ± 8.513	–1252.667 ± 138.399	76,786.667 ± 5707.244	201.833 ± 45.107	–309.267 ± 45.179	964,133.333 ± 46,146.759
		1			37.287 ± 5.346	–1468.333 ± 46.436	275,000.000 ± 31,849.176	136.633 ± 27.618	–173.300 ± 34.282	1,317,666.667 ± 93,948.567
			12.5		21,156.667 ± 4686.196	4695.333 ± 498.740	–5730.333 ± 854.969	21,156.667 ± 4686.196	4695.333 ± 498.740	–5730.333 ± 854.969
			15		8880.667 ± 961.667	–4387.333 ± 716.596	–95,050.000 ± 3558.483	8880.667 ± 961.667	–4387.333 ± 716.596	–95,050.000 ± 3558.483
			17.5		1702.000 ± 187.286	–17,033.333 ± 3442.097	–11,315.667 ± 1472.907	1702.000 ± 187.286	–17,033.333 ± 3442.097	–11,315.667 ± 1472.907
				3	0.000 ± 0.000	0.000 ± 0.000	0.000 ± 0.000	0.000 ± 0.000	0.000 ± 0.000	0.000 ± 0.000
				5	0.000 ± 0.000	0.000 ± 0.000	0.000 ± 0.000	0.000 ± 0.000	0.000 ± 0.000	0.000 ± 0.000
				7	0.000 ± 0.000	0.000 ± 0.000	0.000 ± 0.000	0.000 ± 0.000	0.000 ± 0.000	0.000 ± 0.000
Binary polymeric system	1^a^	0.5	12.5	5	1451.667 ± 167.001	–11 637 ± 1609.265	–6051.667 ± 1120.197	97.953 ± 16.472	–7387.000 ± 591.277	–6219.000 ± 1244.700
	2^a^	1	12.5	5	12,367 ± 1449.1837	–12,413.000 ± 2025.274	21,130.000 ± 3106.381	313.600 ± 10.650	–7770.667 ± 756.176	4796.333 ± 688.585
	3^a^	0.5	17.5	5	1108.333 ± 120.964	–53,393 ± 5409.328	–73,703.333 ± 3532.511	–260.500 ± 27.744	–35,093.333 ± 3495.731	–32,833.333 ± 3208.369
	4^a^	1	17.5	5	44,387 ± 2733.813	–21,550.000 ± 3002.016	–18,323.333 ± 1457.956	464.500 ± 40.444	–44,653.333 ± 8363.506	–51,730.000 ± 9453.618
	5^a^	0.5	15	3	3.248.316.516	–20,340.000 ± 4137.004	–27,946.667 ± 5479.072	–145.233 ± 13.475	–9299.667 ± 1842.730	–7391.000 ± 1389.591
	6^a^	1	15	3	9826.667 ± 620.240	–4400.000 ± 652.800	42,513.333 ± 5911.365	272.033 ± 43.946	–17,530.000 ± 884.251	–4563.000 ± 767.712
	7^a^	0.5	15	7	1213.767 ± 216.016	–39,957.000 ± 4382.880	–110,200.000 ± 15,069.174	276.867 ± 7.336	–25,003.333 ± 1047.107	–16,266.667 ± 1128.819
	8^a^	1	15	7	31,373 ± 3726.8798	–38,073.000 ± 6161.431	48,320.000 ± 8457.358	1223.333 ± 141.550	–26,443.333 ± 4072.595	–7872.333 ± 1316.104
	9^a^	0.75	12.5	3	2778 ± 203.6819	–13,560.000 ± 3808.031	–5517.767 ± 4697.149	164.067 ± 30.601	–2312.667 ± 283.331	–5178.000 ± 912.211
	10^a^	0.75	17.5	3	3691.333 ± 496.643	–25,127.000 ± 3072.301	–23,850.000 ± 4242.8646	263.433 ± 30.104	–30,503.333 ± 5919.268	–50,096.667 ± 2735.038
	11^a^	0.75	12.5	7	3276.333 ± 558.041	–21,510.000 ± 3257.099	21,576.667 ± 4460.452	162.100 ± 15.591	–10,144.333 ± 1161.721	–3847.000 ± 747.785
	12^a^	0.75	17.5	7	5305 ± 274.780	–72,220.000 ± 5280.929	213,233.333 ± 46,383.223	400.000 ± 44.066	–68,483.333 ± 16,121.974	–18,720.000 ± 2857.779
	13^a^	0.75	15	5	3063.000 ± 392.902	–13,863.000 ± 3453.902	–37,480.000 ± 6176.026	–227.100 ± 36.702	–15,750.000 ± 3163.463	–15,093.333 ± 2424.651
	14^a^	0.75	15	5	3897 ± 615.695	–27,703.000 ± 1029.2878	–15,253.333 ± 2437.013	–217.700 ± 34.996	–13,626.667 ± 2181.040	–3444.333 ± 537.986
	15^a^	0.75	15	5	3997.000 ± 566.382	–28,937.000 ± 1707.347	–23,726.667 ± 4426.989	–282.033 ± 37.408	–13,836.667 ± 1886.169	–3127.333 ± 470.358

a Fifteen formulations were prepared using the Box–Behnken 3^3^ design. When acid-extracted collagen (COL) was used, the formulation is called CP. When acid–pepsin-extracted collagen (pCOL) was used, the formulation is called pCP.

The monopolymeric systems at temperature of 10 °C displayed σ_y_ for all collagen concentrations (COL and pCOL) that were not observed for the P407 concentrations. For COL, the concentration of 1% was the most significant (*p* < 0.05), while for pCOL there was no difference between them. At a temperature of 25 °C, the collagen transitioned, losing its influence on the σ_y_ and P407 underwent the micellization process. At a temperature of 34 °C, the increase in the yield value of the monopolymeric system with a high concentration of P407 was evident (*p* < 0.05).

For binary polymeric systems, the combination of collagen (COL or pCOL) and P407 had different responses in the σ_y_ as the temperature increased. At temperature of 10 °C, systems containing the highest concentration of collagen (1%—COL or pCOL) displayed greater influence (*p* < 0.05) compared to low concentrations. When comparing systems with the same concentration of pCOL collagen (1%), the difference was not significant, as it was in systems containing 1% COL collagen. This occurs because in pCOL collagen, pepsin cleaves the terminal chains of telopeptides, which leads to a decrease in the viscosity donor power and facilitates its solubility. In both the system containing COL and pCOL, significant σ_y_ values were verified in the system containing 1/17.5/5% (CP4 and pCP4—collagen/P407/Gly). At 25 °C, the concentrations of 0.75 and 1% collagen displayed influence; however, the formulations with the highest concentrations of P407 stood out. At 34 °C, the formulations with the highest concentrations of P407 were significant (*p* < 0.05). This characteristic was common in the systems containing COL and pCOL collagen, for both systems, with significant σ_y_ for the formulations 0.75/17.5/7% (CP12 and pCP12—collagen/P407/Gly). High σ_y_ favor the applicability of the semisolid pharmaceutical form, since a higher shear stress will be necessary for the systems to flow and, thus, their retention at the applied site is increased.

It is noted that with the increase in temperature and polymer concentration, there was a significant increase in the σ_y_. The highest σ_y_ were observed at a temperature of 34 °C in the presence of P407 at the highest concentrations. When analyzing the binary polymer systems, it was observed that Gly at the highest concentration (7%) increases the viscosity of the system and the highest concentration of collagen weakens the hydrophobic interactions between the P407 chains responsible for thermal gelation, as seen previously.^83^ This statement can be confirmed by comparing the σ_y_ and *K* values of the entire binary polymer system at 34 °C, since formulations containing 0.75/17.5/7% and 0.75/17.5/3% (COL or pCOL/P407/Gly) displayed significant difference (*p* < 0.05). Just as the higher concentration of collagen COL 1/15/7% (CP8) versus 0.5/15/7% (CP7) and 1/15/3% (CP6) versus 0.5/15/3% (CP5) (COL/P407/Gly) could reduce the *K* values and σ_y_ with a significant difference (*p* < 0.05). For collagen pCOL the difference was not so significant, since the removal of telopeptides reduced the viscosity of the system, making the concentration of P407 and glycerin the variables that affect the *K* values and σ_y_.

The rheological behavior evaluated at different temperatures provided indirect evidence of thermal gelation. The increase in *K* and σ_y_ observed at 34 °C, especially in formulations with higher concentrations of P407, suggests the formation of a gel-like structured network at physiological temperatures. This behavior is consistent with the known thermoresponsive properties of P407, which undergoes micellar packing and transition to gel above its critical micellization temperature. These rheological changes are relevant both for injectable systems, where gelation near body temperature (33–37 °C) allows the formation of an in situ deposit, and for topical formulations, where increased viscosity favors retention at the application site. Therefore, although *T*_sol/gel_ was not measured directly, the rheological profiles observed in this study are consistent with the transition behaviors described in the literature for poloxamer-based systems.

When subjected to shear stress, some materials undergo a reversible loss of viscosity that may present hysteresis. The hysteresis area is a way of describing the response of the material as a function of shear stress over time. Upon reaching the maximum shear stress and time, the process is reversed by decreasing shear stress, resulting in a region delimited by the upward and downward curves (Figures S1–S6). The hysteresis area may present thixotropic or rheopectic profile. The representation of the hysteresis area can be given in micrographs of shear stress versus shear rate (where thixotropy is represented when the downward curve is smaller than the upward curve and rheopectic occurs when the downward curve is higher than the upward curve) and/or in numerical values (thixotropic—positive hysteresis area or rheopectic—negative hysteresis area) (Table 6).

At a temperature of 10 °C, the most binary polymer systems presented thixotropy, which means that the viscosity of the system decreases when agitated and applied; and increases again when at rest, in a time-dependent way, which facilitates application and improves adhesion to the skin, then gradually recovers as the tension is removed. Considering that at this temperature the highest concentration of collagen was significant (*p* < 0.05), it is understood that with the increase in the shear rate, the aggregates dissociate into monomers, and the previously rolled and entangled collagen molecules straighten along the direction of the shear force, facilitating the flow of collagen solutions (i.e., decreasing viscosity).^87^ In a thixotropic material, the molecular structure or particle organization changes from a more viscous form to a less viscous form over time when subjected to stress, improving dosage uniformity, prolonging contact time, and facilitating adhesion to skin and mucous membranes.^76,88^ Some vaccine and adjuvant formulations can be designed to exhibit thixotropic behavior to improve vaccine delivery and efficacy by enabling controlled release of active ingredients.^89,90^

As the temperature increased from 10 to 25 °C, all binary polymeric systems displayed a tendency to change the behavior from thixotropic to rheopectic. This phenomenon occurred because with the increase in temperature, P407 began the micellization process; this can be confirmed by analyzing the P407 monopolymer system. The rheopectic characteristic has already been reported in colloidal dispersions, as a reversible phenomenon with a time-dependent increase in viscosity when exposed to a certain shear rate, that is, rheopectic systems become more viscous. A practical example can be found in ophthalmic formulations.^91,92^ Some gels or eye drops may be formulated to increase their viscosity after application to the eye. This may prolong the residence time of the drug in the eye, thereby improving the efficacy of the treatment. Some topical ointments and gels may be formulated to exhibit rheopectic hysteresis. This may be beneficial in prolonging the contact time of the drug on the skin, thereby improving absorption and providing longer adhesion. At 34 °C, some binary polymer systems (both COL and pCOL) displayed thixotropy. The difference among the hysteresis areas of formulations at this temperature shows how the variables responded differently depending on the concentration of each component of the formulation.

The choice of the best formulation depends largely on the application site and the way in which the product will be applied. Continuous flow rheological analysis may involve investigating how materials behave under different shear, temperature, and concentration conditions, among other factors. This is particularly relevant in many industrial sectors, such as food, cosmetics, pharmaceuticals, and polymers, where the ability to understand and control the continuous flow of materials is crucial for product development and manufacturing, providing valuable information to optimize industrial processes and ensure product quality. The binary polymer systems of collagen (COL and pCOL) and P407 at different temperatures and concentrations allow us to apply these systems for different purposes, showing the importance of better understanding this system to provide a system with quality, safety, and efficacy. Thus, in the following analysis, we will understand how the binary polymer systems in this study behave when faced with oscillating forces instead of constant shearing forces.

### Oscillatory Rheological Analysis

Oscillatory rheology plays a significant role in the characterization of polymeric systems, regarding their structure and behavior during preparation, transportation and use. It provides detailed information on how polymers respond to oscillatory forces, which is crucial to understanding their viscoelastic properties. The storage or elastic modulus (*G*′) is related to the polymer’s ability to store energy during elastic deformation. The loss or viscous modulus (*G*″) reflects the dissipation of energy during viscous deformation. The relationship between *G*′ and *G*″ provides information on the elastic or viscous dominance of the material under different conditions, such as at the application site and during storage. It also helps us understand the mechanical properties that affect the interaction with biological tissues.

The effects of temperature and polymer concentration on the oscillatory properties (*G*′, *G*″, η′ and tan δ) of the monopolymeric collagen preparations (COL and pCOL) and P407 at 13 representative oscillatory frequencies are displayed in Figures 6–8.

**Figure 6:**
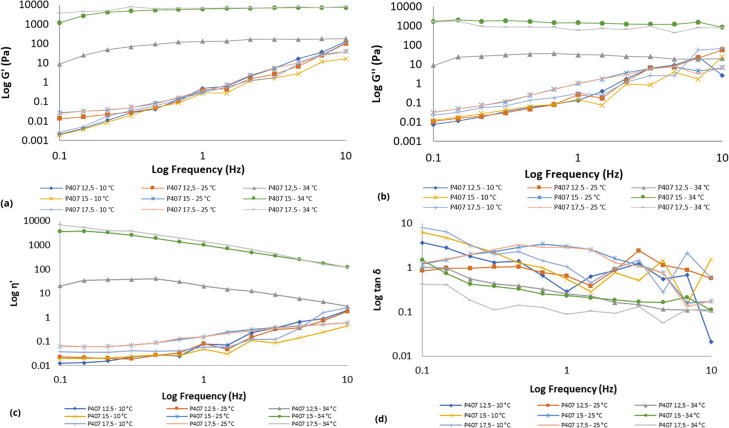
Viscoelastic properties as a function of frequency of monopolymeric formulations of poloxamer 407 (P407), at temperatures of 10, 25, and 34 °C: (a) elastic modulus (*G*′); (b) viscous modulus (*G*″); (c) dynamic viscosity (η′); (d) loss tangent (tan δ). Each graph is the average of at least three replicate samples.

**Figure 7:**
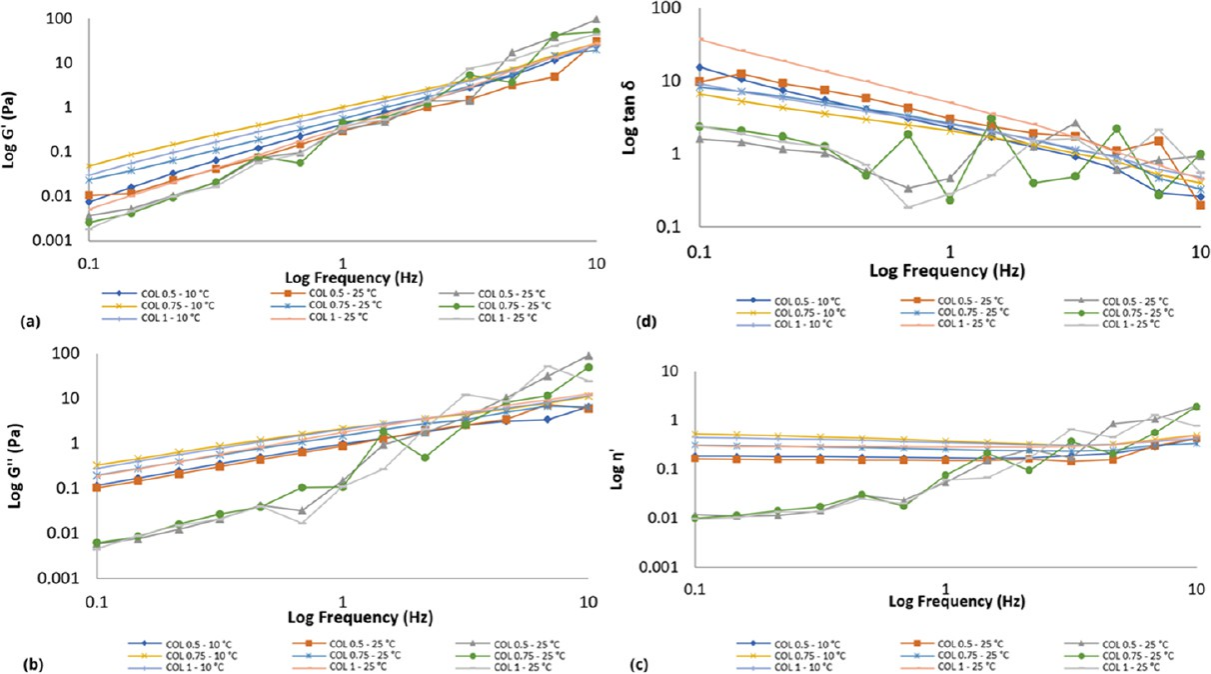
Viscoelastic properties as a function of frequency of monopolymeric formulations of collagen obtained by acid extraction (COL), at temperatures of 10, 25, and 34 °C: (a) elastic modulus (*G*′); (b) viscous modulus (*G*″); (c) dynamic viscosity (η′); (d) loss tangent (tan δ). Each graph is the average of at least three replicate samples.

**Figure 8:**
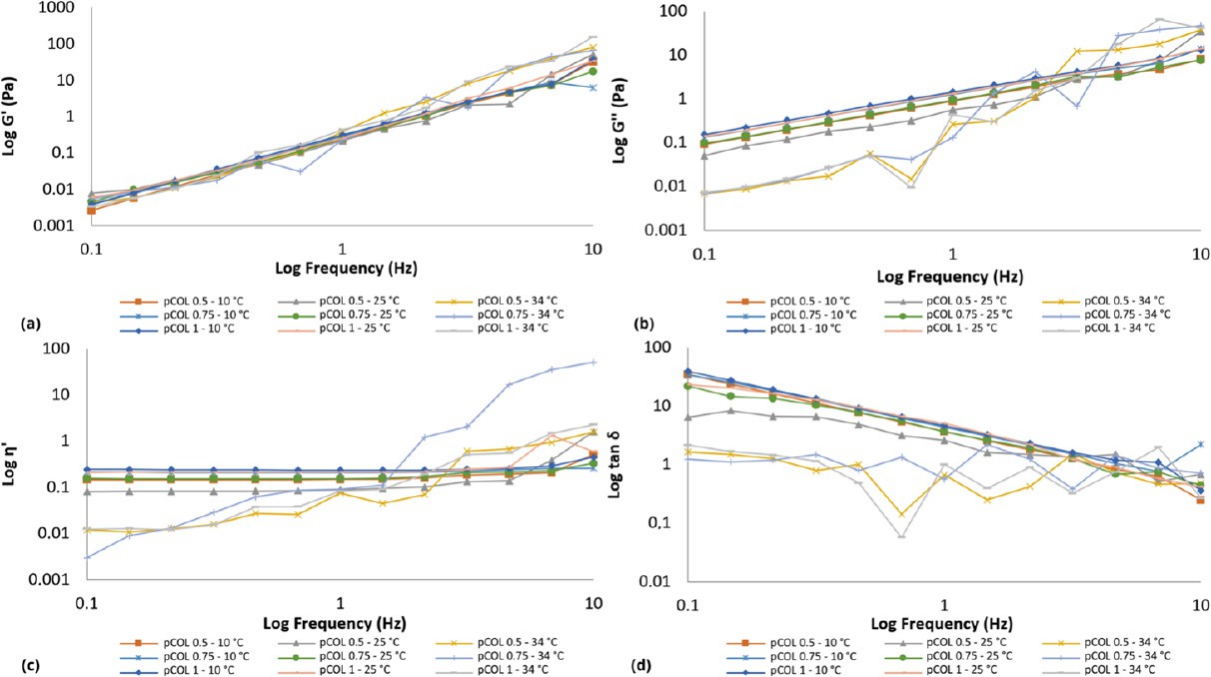
Viscoelastic properties as a function of frequency of monopolymeric formulations of collagen obtained by acid extraction with pepsin (pCOL), at temperatures of 10, 25, and 34 °C: (a) elastic modulus (*G*′); (b) viscous modulus (*G*″); (c) dynamic viscosity (η′); (d) loss tangent (tan δ). Each graph is the average of at least three replicate samples.

For the P407 monopolymer solution, the *G*′ and *G*″ values were too low to obtain reliable measurements at a temperature below 25 °C, indicating that the sample remains in a liquid state. When analyzed at a temperature of 34 °C, it is noticeable that the P407 monopolymer system passes to the gel state due to the increased values of *G*′ and *G*″. Similar results were found in the study by Fu and collaborators.^83^ The same occurred in monopolymer formulations of COL and pCOL collagen at a temperature of 34 °C. In general, the monopolymer systems had their *G*′, *G*″ and η′ values influenced by temperature, polymer concentration and frequency.

The loss tangent (tan δ) is a measure used in oscillatory rheology analysis to describe the relationship between the viscous and elastic parts of a material’s viscoelastic behavior. The loss tangent is calculated as the ratio between the *G*″ and the *G*′ at a given point during an oscillatory rheology test. The result of this division results in values greater or less than 1 (one). Values above 1 characterize elastoviscous systems, which have *G*″ values greater than *G*′. On the other hand, when the values of tan δ are less than 1, the systems have viscoelastic properties, this means that *G*′ is greater than *G*″.

In this sense, the monopolymeric systems based on P407 at concentrations of 12.5, 15 and 17.5% at 34 °C presented viscoelastic properties, while at 10 and 25 °C they presented elastoviscous characteristics (Figure 6). As the frequency increased, the polymeric systems at 10 and 25 °C presented an increase of *G*′, *G*″ and η′. At 34 °C, the values of *G*′ and *G*″ were higher than at the other temperatures; however, the increase in frequency did not significantly increase *G*′ and *G*″; and decreases η′. Since *G*″ predominates at 10 and 25 °C, it is clear that the P407 micelles are not packed in an ordered manner and that the polymer chains are probably in a flexible conformation. But at 34 °C, the rapid rise in *G*′ represents the development of a strong gel network with *G*′ > *G*″ regardless of the oscillatory frequency. Similar results were found in studies evaluating the effect of frequency on *G*′, *G*″ and η′ at different P407 concentrations at different temperatures.^53,93^

The monopolymeric systems based on COL and pCOL collagen showed elastoviscous characteristics, meaning that *G*″ is greater than *G*′, except for the concentrations analyzed at 34 °C, which presented values too low to obtain reliable measurements (Figures 7 and 8). When comparing the monopolymeric systems containing different types of collagens, we found that the *G*′ of COL is greater than that of pCOL, suggesting that intact telopeptides contribute to increased elasticity of the formulations, improving interactions between collagen molecules. In order to better elucidate the difference between the two types of collagens obtained from rat tails (with and without telopeptides), Shayegan and collaborators evaluated whether this change in viscoelasticity is related to the difference between the types of commercial samples. Therefore, the authors found that the removal of telopeptides reduces elasticity and viscosity as pepsin cleaves them. Furthermore, they found that it was the activity of pepsin and not its presence in the sample that altered the complex shear modulus.^94^

Overall, oscillatory frequency, temperature, and polymer concentration affected the viscoelastic properties of binary systems containing P407 and collagen (COL or pCOL) (Figures 9–16). The increase in oscillatory frequency resulted in an increase in *G*′ and *G*″, as well as a decrease in η′ and tan δ. At 10 °C, binary systems with the highest collagen concentration (1%—COL or pCOL) showed the highest *G*′ and *G*″ values. Whereas at 25 °C, systems with the highest P407 concentration started to increase. At 34 °C, systems containing 0.5/17.5/5; 0.75/17.5/3, and 0.75/17.5/7 (CP3; CP10 and CP12—COL/P407/Gly) showed the highest *G*′ and *G*″ and η′ values. For the binary polymer systems containing COL, in addition to the concentrations described above (pCP3; pCP10 and pCP12), the system containing 1/17.5/5 (pCP4—pCOL/P407/Gly) presented the highest values of *G*′ and *G*″ and η′, indicating that cleavage of the telopeptide does not alter the hydrophobic interactions between the P407 chains, as occurs in collagen containing COL.

**Figure 9:**
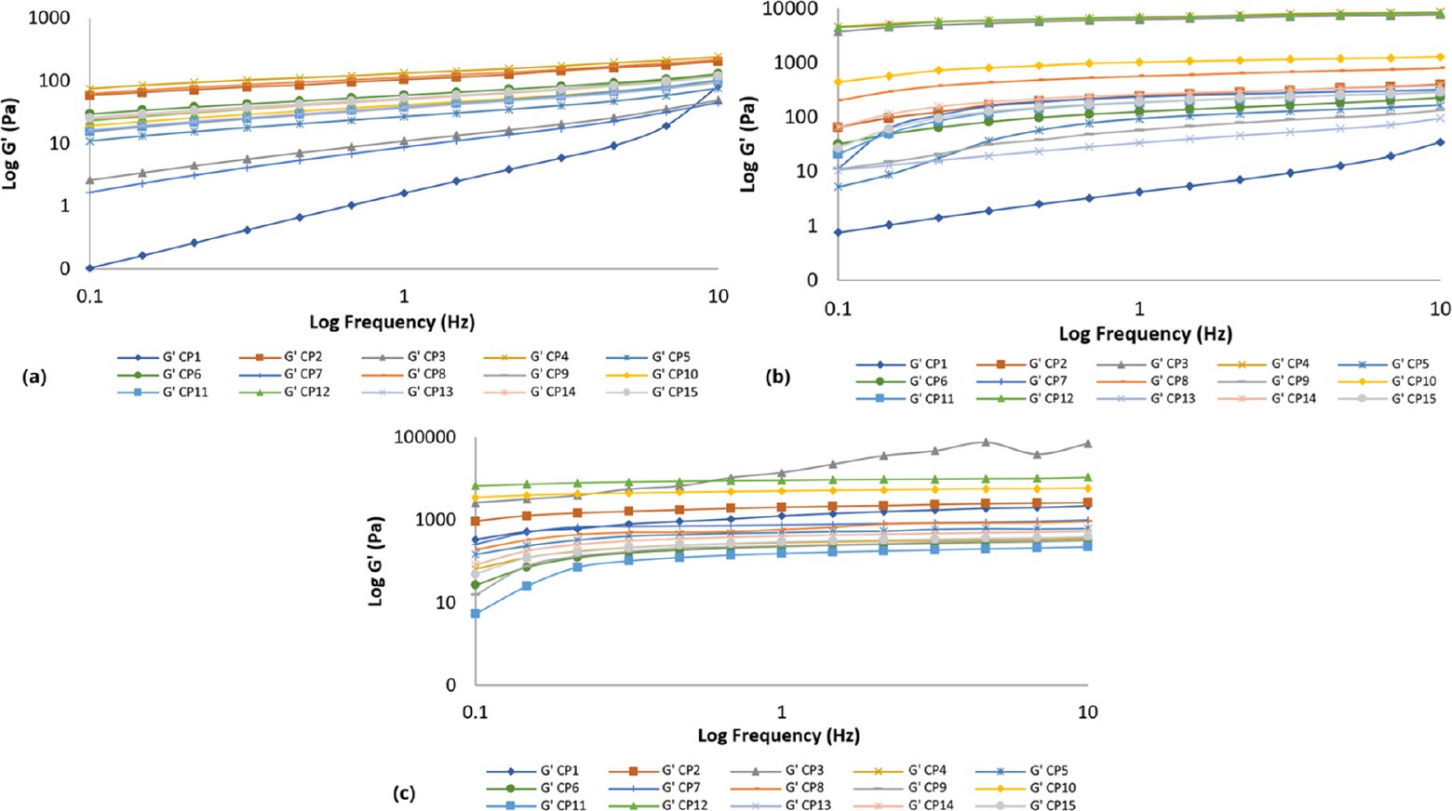
Elastic modulus (*G*′) as a function of frequency for binary polymer formulations containing collagen obtained by acid extraction (CP) at temperatures of: (a) 10 °C; (b) 25 °C; (c) 34 °C. Each graph is the average of at least three replicate samples.

**10:**
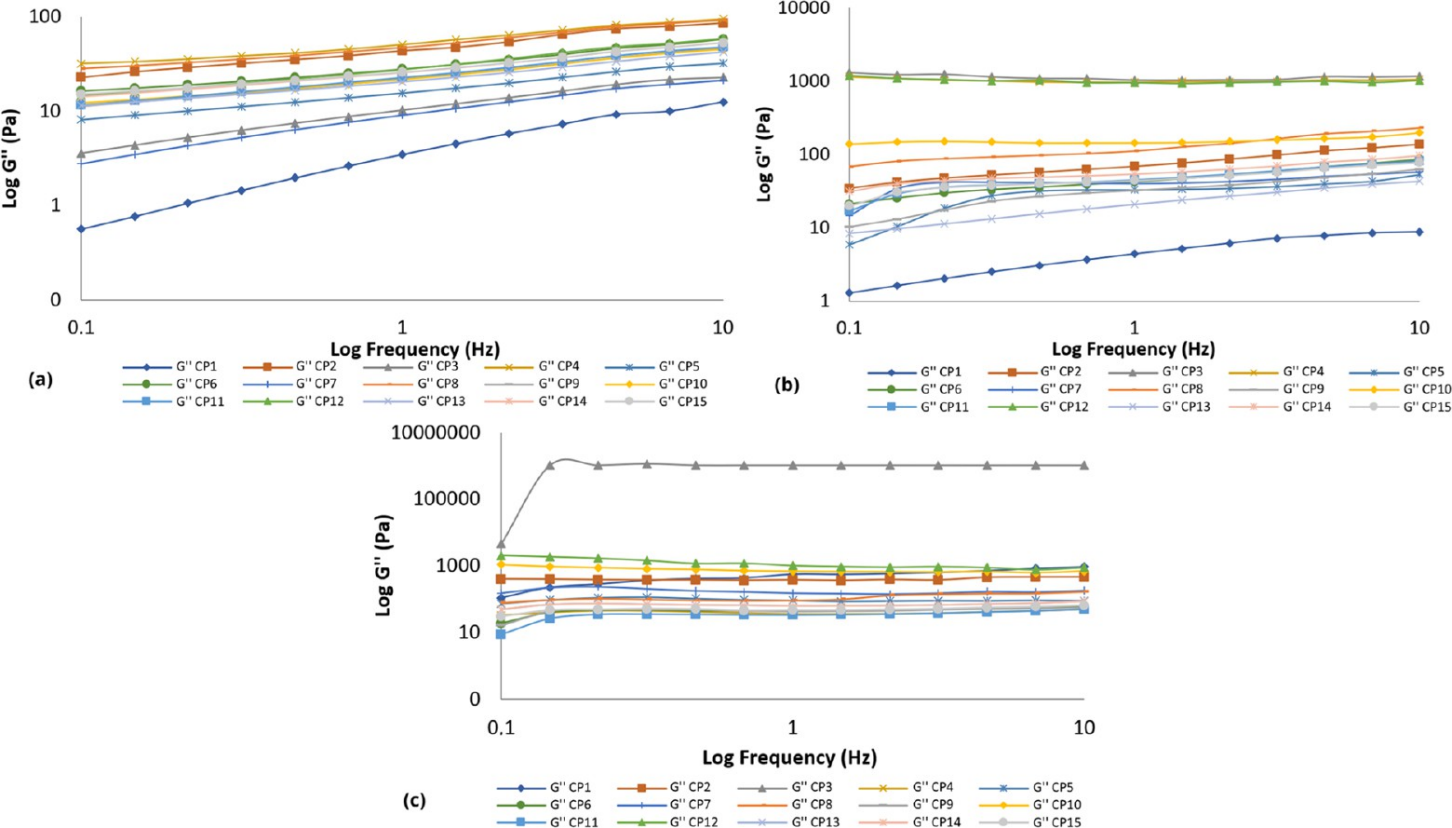
Viscous modulus (*G*″) as a function of frequency of binary polymer formulations containing collagen obtained by acid extraction (CP) at temperatures of: (a) 10 °C; (b) 25 °C; (c) 34 °C. Each graph is the average of at least three replicate samples.

**11:**
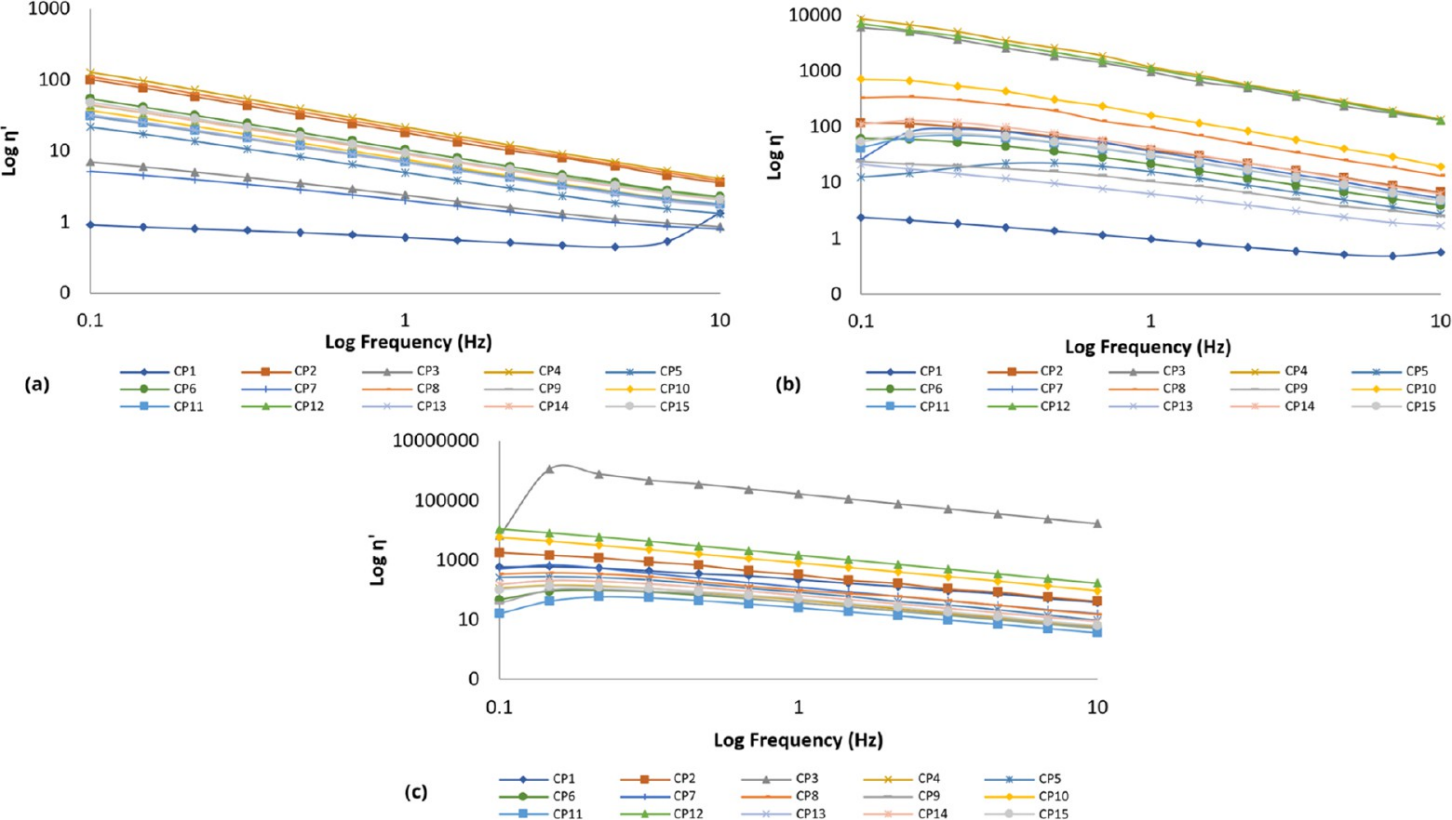
Dynamic viscosity (η′) as a function of frequency for binary polymer formulations containing collagen obtained by acid extraction (CP) at temperatures of: (a) 10 °C; (b) 25 °C; (c) 34 °C. Each graph is the average of at least three replicate samples.

**12:**
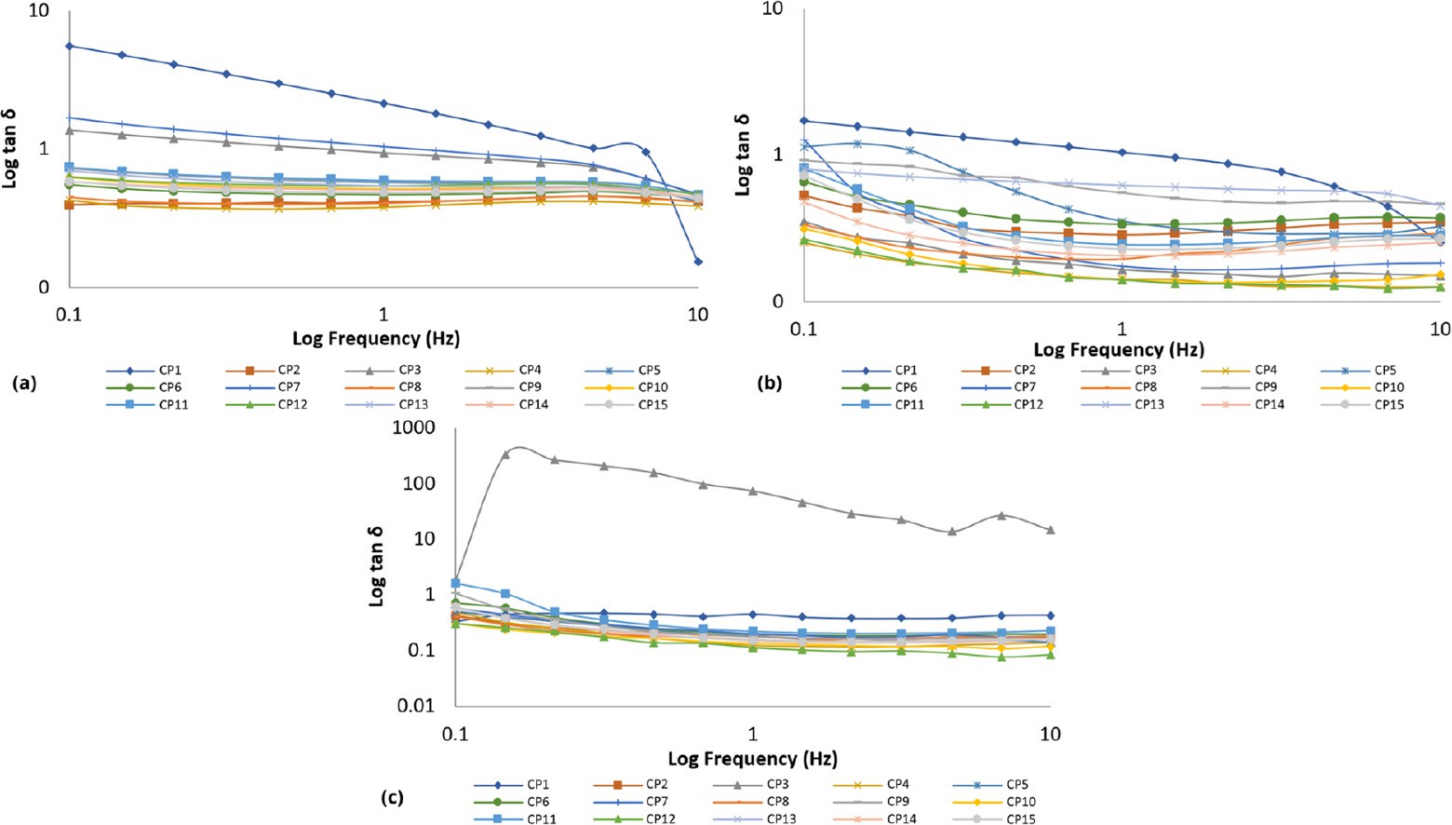
Loss tangent (tan δ) as a function of frequency for binary polymer formulations containing collagen obtained by acid extraction (CP) at temperatures of: (a) 10 °C; (b) 25 °C; (c) 34 °C. Each graph is the average of at least three replicate samples.

**13:**
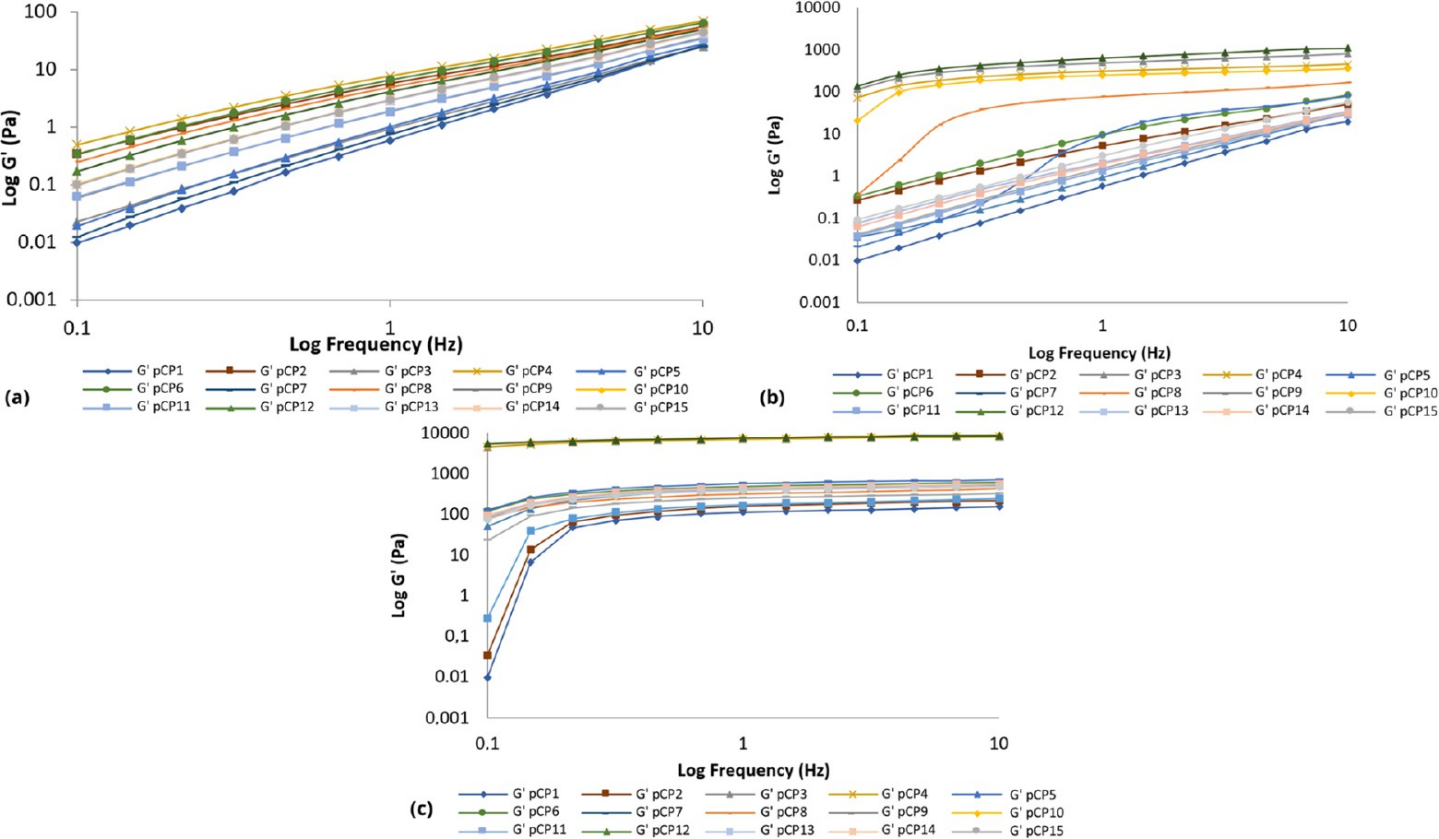
Elastic modulus (*G*′) as a function of frequency for binary polymeric formulations containing collagen obtained by acid extraction with pepsin (pCP) at temperatures of: (a) 10 °C; (b) 25 °C; (c) 34 °C. Each graph is the average of at least three replicate samples.

**14:**
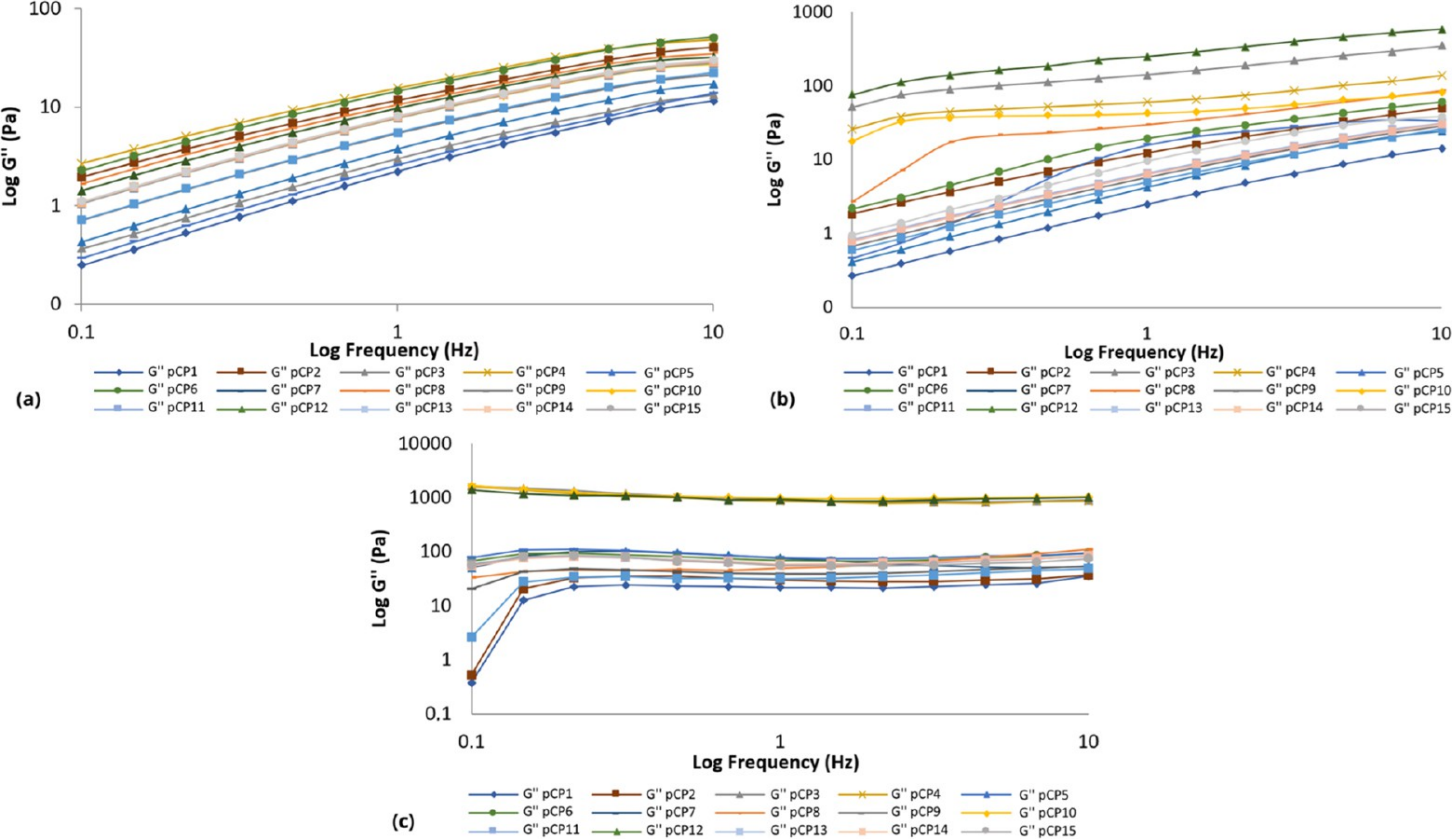
Viscous modulus (*G*″) as a function of frequency for binary polymeric formulations containing collagen obtained by acid extraction with pepsin (pCP) at temperatures of: (a) 10 °C; (b) 25 °C; (c) 34 °C. Each graph is the average of at least three replicate samples.

**15:**
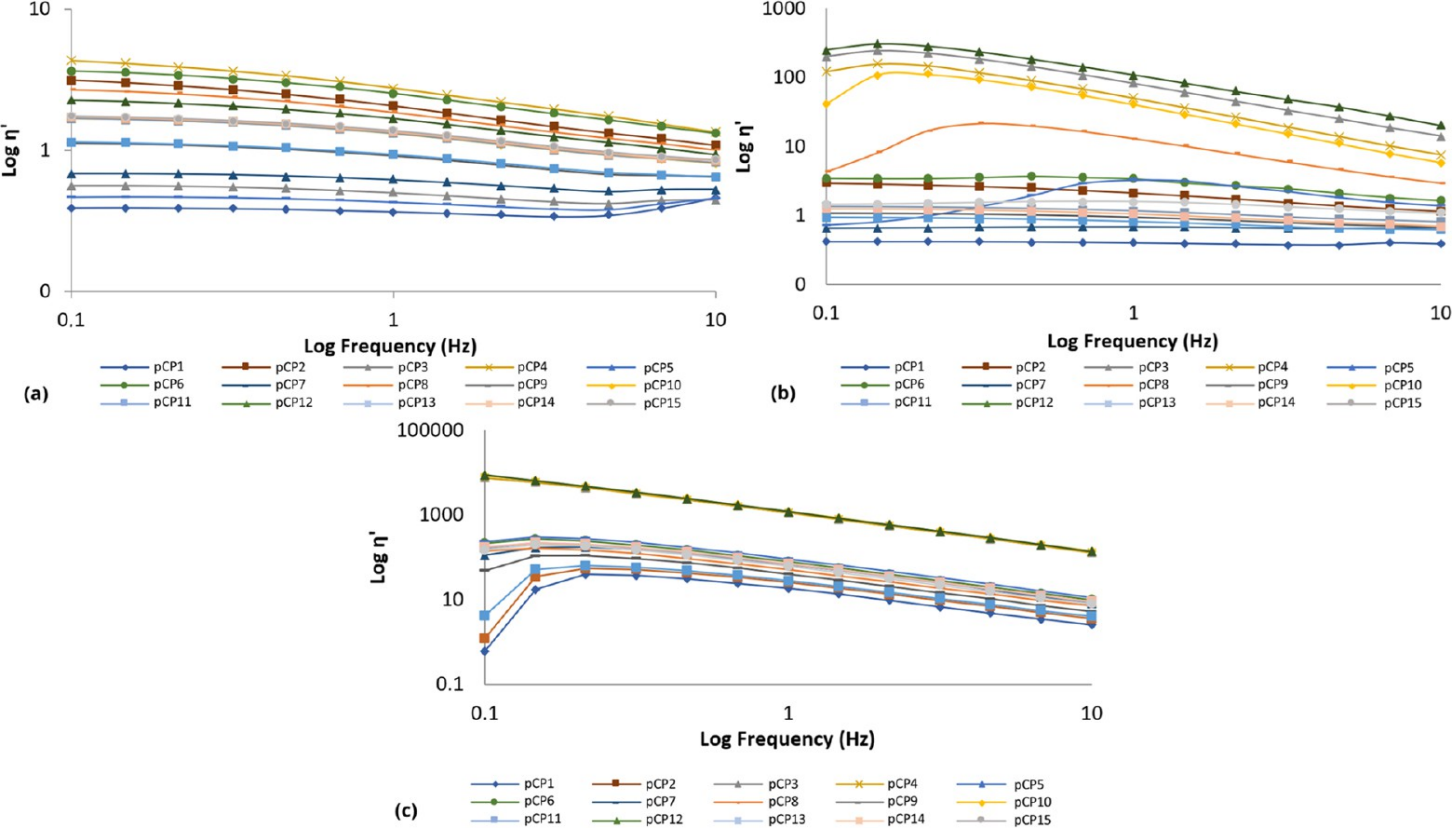
Dynamic viscosity (η′) as a function of frequency for binary polymer formulations containing collagen obtained by acid extraction with pepsin (pCP) at temperatures of: (a) 10 °C; (b) 25 °C; (c) 34 °C. Each graph is the average of at least three replicate samples.

**Figure 16:**
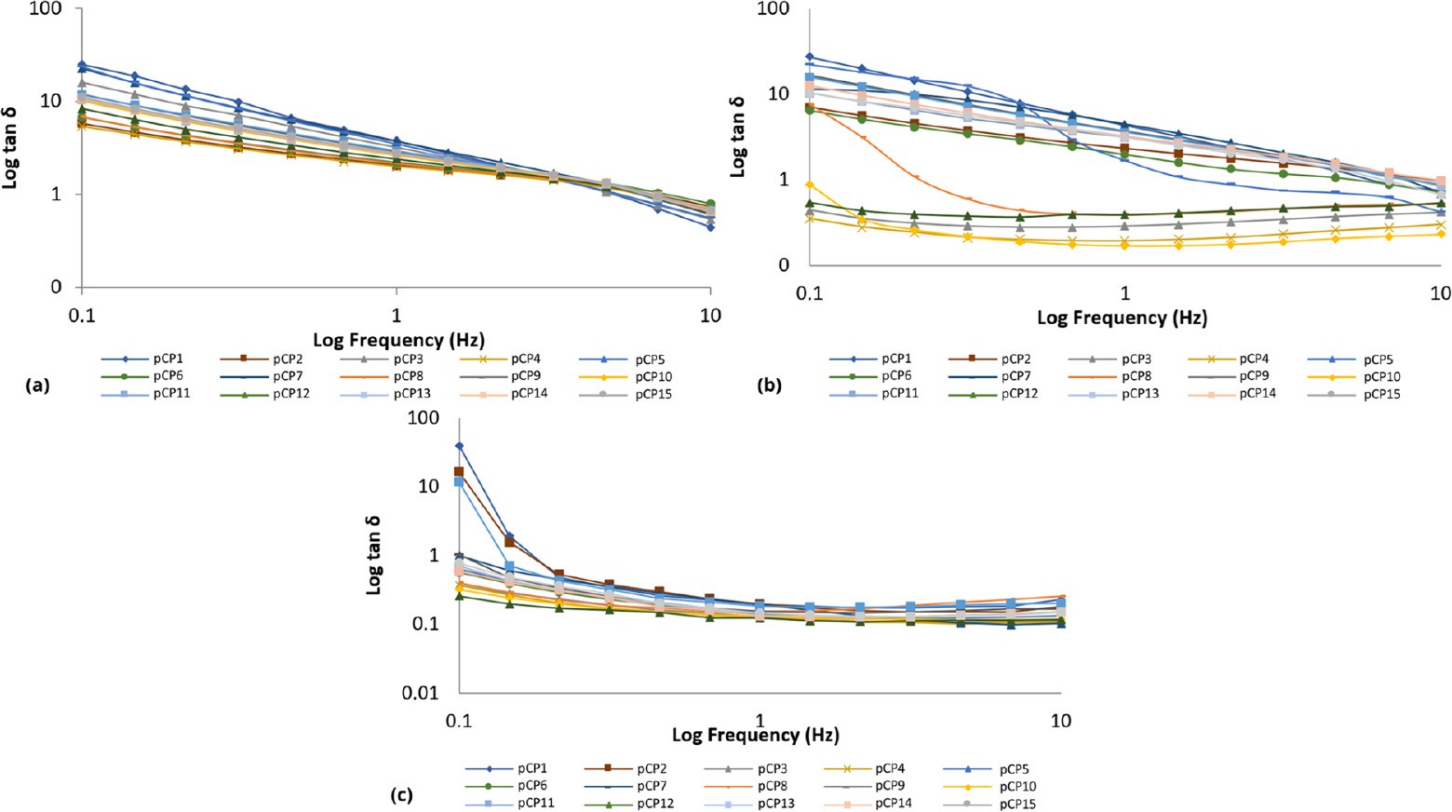
Loss tangent (tan δ) as a function of frequency for binary polymer formulations containing collagen obtained by acid extraction with pepsin (pCP) at temperatures of: (a) 10 °C; (b) 25 °C; (c) 34 °C. Each graph is the average of at least three replicate samples.

Most binary polymer systems composed of COL collagen and P407 presented viscoelastic properties with the exception of the systems containing 0.5/17.5/5% and 0.5/15/7% (CP3 and CP7—COL/P407/Gly) (10 °C); 0.5/12.5/5% (CP1—COL/P407/Gly) (10 and 25 °C) and 0.5/17.5/5 (CP3—COL/P407/Gly) (34 °C). In contrast, the binary polymeric systems composed of collagen pCOL and P407 presented differentiated properties as the temperature was changed. At 10 °C, the entire system presented elastoviscous properties and at 34 °C, the entire system presented viscoelastic properties. At 25 °C, the systems pCP3, pCP4, pCP10 and pCP12 presented viscoelastic properties and the remainder presented elastoviscous properties. The cleavage of the telopeptide interferes with the viscoelastic properties of the systems and it is noted that the properties of the binary polymeric systems are very similar to those of the monopolymeric systems. That is, at low temperatures, P407 is unable to form micelles, and collagen begins to have a greater influence, leaving the entire binary polymer system containing pCOL collagen with elastoviscous properties. At the highest temperature (34 °C), P407 begins to have a greater influence on the binary polymer system, therefore with elastoviscous properties. And at 25 °C, formulations with the highest concentration of P407 (17.5%) presented viscoelastic properties.

This viscoelastic behavior is caused by the supramolecular structure of P407 formed by high temperatures and physical interactions. P407 probably self-organizes into micelles and then the micelles form a three-dimensional network that is stabilized by the interaction of the hydrophilic shell with the collagen chains. The long collagen chains ensure the connectivity of the network and, therefore, the gel is stable at low and moderate values of deformation or shear stress, being beneficial for several applications (injectable gels or topical hydrogels).^95^ In addition, the rheological behaviors of hydrogels, pointed out as typical characteristics of a hydrogel, can present a typical fluid behavior (*G*″ > *G*′) at low temperatures and as the temperature increased, *G*′ increased and eventually crossed *G*″, occurring a process called “crossover”.

The binary systems studied displayed improved physicochemical and rheological characteristics useful for biomedical and pharmaceutical applications. In case of application in mucous membranes and skin, the formulations containing collagen at 1% COL; 17.5% P407 and 5% Gly (CP4) and 0.75% COL; 17.5% P407 and 7% Gly (CP12) can display well-calibrated rheological characteristics capable of withstanding high shear rates and dilution of tears, saliva and sweat, preventing the drugs from being drained from the absorption point. The CP4 and CP12 formulations present perfect characteristics for pharmaceutical formulations that require or do not require refrigeration. In the physiological condition, pseudoplasticity, that is, a decrease in viscosity with increasing shear rate (physiological movements) is a desirable phenomenon for uniform distribution of the hydrogel over a desired surface in response to movement.^93^

Smart gel systems, composed of P407, spontaneously form a network in response to external stimuli, such as temperature. This characteristic of this type of gel is an innovation in the search for new drug delivery systems, including those intended for parenteral applications. Systems with concentrations above 15% P407 undergo a reversible thermal transition from easy-to-handle micellar liquids to shear-thinning physical gels, making them ideal for injectable drug delivery applications.^96^ The combination with collagen provides these systems with adequate porosity, which allows the drug to be carried to the application site.^97^ For the application of injectable formulations, it is essential to observe the viscoelasticity of the system, as well as the hysteresis area, so that the system flows through the needle. Due to the ease of incorporating collagen, systems containing pCOL collagen are the most recommended (pCP4; pCP6; pCP8; pCP10 and pCP12).

The polymeric interaction between collagen and poloxamer 407 (P407) in binary systems can be interpreted as a combination of supramolecular physicochemical forces that result in the modulation of the three-dimensional structure of the polymer matrix. Collagen, a structural protein with a high density of functional groups (particularly hydroxyls, carboxyls and amines)^44,98,99^ has the potential to establish electrostatic or hydrogen bonds with the hydrophilic ethylene oxide (PEO) blocks of P407.^100–102^ Although specific studies on the interaction between collagen and poloxamer 407 are still limited, it is possible to suggest, based on evidence from similar systems involving gelatin, that noncovalent interactions may occur between these macromolecules. This potential affinity may allow the anchoring of collagen molecules on the surface of P407 micelles, which organize themselves with increasing temperature. This behavior suggests a costabilization effect or even a form of noncovalent physical cross-linking between the polymers, which could contribute to the integrity and resistance of the gel.^101^ Furthermore, the amphiphilic nature of P407 allows the formation of micelles with hydrophobic core and hydrophilic corona, where collagen could act as a compatibilizing agent at the micellar interface, promoting additional structural interactions. This configuration could contribute to a reinforcement of the micellar network and to the formation of a more robust and elastic gel system, which is suggested by the increase in the storage modulus (*G*′) in the formulations containing collagen. From a rheological point of view, this more structured arrangement may be related to the observed pseudoplastic behavior, at rest or under low shear rates, the network of interactions between collagen and P407 keeps the system more viscous and stable. However, as the material is subjected to mechanical stress (such as during extrusion by syringe or withdrawal from the system of a packaging material) these interactions reorganize, facilitating flow. This shear-thinning behavior is especially desirable both for in situ injectable applications and for emerging technologies such as PAM. In these cases, the formulation needs to maintain sufficient viscosity to remain stable at rest, but it also needs to flow easily when pushed through a syringe, resuming its structured shape immediately after deposition.

Additionally, the observed molecular and behavioral interactions indicate that glycerin not only acts as a plasticizer, increasing the viscosity and elasticity of formulations, but also plays a crucial role in modulating the gel structure. The presence of glycerin in the system can influence the solution-to-gel transition, reducing the rigidity of polymer networks and providing greater flexibility and thermal stability. Furthermore, glycerin can promote hydrophobic and hydrophilic interactions between collagen and P407 chains, facilitating the formation of a more uniform and resilient structural network. These synergistic effects contribute to improved rheological properties, adjusting the system’s viscoelasticity and conferring ideal characteristics for applications in tissue engineering and controlled drug release. In particular, glycerin’s ability to increase viscosity and modify the system’s elasticity is essential for optimizing deposition stability and precision during 3D printing, in addition to providing a controlled and prolonged release of the therapeutic active.

Furthermore, the use of hydrogels goes beyond topical or parenteral application, with the possibility of printing 3D constructs for biomedical and pharmaceutical applications. There are different types of 3D printers, each using a different type of ink to obtain a printed structure.^103^ Rheology can offer a useful tool to obtain reproducible information about ink properties, which are important for printing. Each ink has a rheological particularity that is functional for what is intended to be printed. For this reason, different rheological models, such as the power law, Casson, and Herschel–Bulkley, are widely used to describe the behavior of fluid flow during the printing process.^104–108^ Important material-specific parameters such as *K*, *n*, σ_y_ and viscoelastic behavior of the bioink can be extracted from the rheological data to determine the shear stress condition at the nozzle during the printing process.

When developing ink for 3D printing, many factors must be evaluated, especially if the purpose is tissue engineering. In these cases, during the bioprinting process, there are many factors that can influence cell survival, such as shear force at the printing nozzle; printing temperature; access to nutrients and oxygen; nozzle outlet diameter; and cell type present in the bioink.^103^ During the printing process, when extruding the ink, there is a transition point where the ink flows vertically out of the syringe to be dragged onto the fixed printing plate. At this transition point, viscoelastic stresses are accumulated at the end of the nozzle (tip of the printing syringe needle), so the stress is greater and tends to decrease as the ink is extruded.^108^ To obtain a continuous line of printed ink, the pressure must be sufficient to dispense a continuous thread at the given speed. If the pressure applied to the printing syringe is too high, the width of the hydrogel thread will become larger than the width of the printing nozzle.^107^

Most studies involving the use of collagen-based inks for 3D printing report their low mechanical properties as the main problem in the use of this ink. One approach to overcome this limitation is the use of support polymers in the hydrogel. The copolymer P407 was consciously incorporated into the tilapia skin collagen hydrogel. The good solubility in water and alcohol, and the thermoresponsiveness of P407 (solution at low temperatures and gel at a temperature of approximately 35 °C) give it good characteristics that benefit 3D printing inks.^105^ This binary combination in the system resulted in binary polymer systems with improved mechanical and rheological properties, as seen previously.

Another way to increase the printability of collagen-based inks is to increase the storage modulus of the ink prior to extrusion.^109,110^ In other words, it has been shown that collagen-based inks with a storage modulus much higher than the loss modulus are suitable for direct extrusion 3D printing. Therefore, the predominance of the loss modulus means that the ink behaves like a fluid with insufficient storage modulus to maintain the shape of the printed structure. The authors observed that the pore diameter of the printed material became smaller, and the filament width became wider compared to the intended extrusion conditions, concluding that the ink still lacked the storage modulus necessary to hold the structure together and reduce material flow.^111^

To increase the storage modulus, a higher concentration of collagen in the bioink and heating of the hydrogel to 37 °C have been suggested.^110^ In a study with collagen-based bioink in the fabrication of scaffolds, it was found that the scaffold with the best structure were those printed at a plate temperature between 36 and 39 °C.^70^ The choice of the best binary polymer system based on collagen and P407 will depend on its purpose. The biofabrication of scaffolds, bones, cartilage or constructs that require maintaining the structure horizontally requires a material with a high *K* value and that is pseudoplastic and thixotropic, in addition, the storage modulus must be much higher than the loss modulus. Moreover, pharmaceutical films are a thin, solid dosage form that can contain drugs and are designed to be administered in a specific manner, usually orally, buccally, transdermally, or by other routes.^112^ These films are often used as convenient and innovative alternatives to traditional forms of drug delivery. Although they do not have a standard thickness, in general, pharmaceutical films usually have a thickness in the range of micrometers (μm) to a few millimeters.^113^

Pseudoplasticity is an essential property for materials intended for 3D extrusion printing, as it allows the material to temporarily reduce its viscosity under the application of force and return to a more structured state after deposition.^114,115^ In addition, another equally important criterion for this application is the shape recovery capacity, that is, the recovery of rigidity immediately after extrusion, which ensures that the material maintains its printed shape faithfully. The data obtained in this study demonstrate that, unlike the isolated collagen or P407 monopolymer systems, the binary formulations exhibited pseudoplastic behavior at all temperatures analyzed, indicating a polymeric interaction that modifies the mechanical response of the system. Furthermore, not only was robust pseudoplastic behavior observed in the binary mixtures, but also the presence of a σ_y_ in the rheological profiles, suggesting that these formulations are capable of withstanding initial deformations and then recovering structural cohesion.^116^ This behavior suggests that these formulations meet the minimum rheological criteria required for extrusion 3D printing applications, especially regarding extrusibility and postdeposition shape recovery. In a study with printing ink based on bovine collagen, it was noted that it presented a pseudoplastic profile favorable to pneumatic extrusion, allowing the printing of stable structures.^117^ Gelatin–alginate–hyaluronic acid-based inks have demonstrated favorable shear-thinning behavior, being effective in printing scaffolds with good definition and structural stability.^118^ This shear-thinning behavior is so critical for printing success that specific strategies have been adopted to induce or optimize it. For example, oil-based support baths, such as those containing fumed silica in mineral oil, have been used to impart shear-thinning behavior to the support matrix and control rheology during the printing of pH-sensitive hydrogels.^119,120^ This type of approach highlights how rheological modulation, especially shear-thinning, is a central prerequisite in the development of effective bioinks for 3D printing of functional hydrogels.

Although we did not perform direct printing tests in this study, the rheological results presented provide support for considering the viability of these formulations in syringe-based bioprinting systems, as a starting point for future experimental validations. In preliminary studies, it was found that the high concentration of P407 interferes with film formation, making it more brittle. Considering the rheology and ease of film formation by hydrogels, the CP2 and CP8 formulations could be utilized as film-form systems. Although at 10 °C the systems containing pCOL are not viscoelastic, the pCP2 and pCP8 systems will also be printed, since the removal of these peptides results in collagen known as atelocollagen, which has a lower capacity to form organized fibrils and lower thermal stability. In addition, pepsinized collagen has a lower viscosity that favors its manipulation in advanced technological processes, such as 3D extrusion printing (PAM).

### SEM Analysis

SEM morphological analysis revealed that variations in the concentration of P407 and glycerin, as well as the type of collagen used (native or pepsinated), significantly influence the microstructure of the hydrogels (Figure 17). Systems CP2 and pCP2, obtained with lower concentrations of poloxamer and glycerin, presented a more open and highly porous network, with greater interconnectivity between the pores. This characteristic favors cell adhesion and nutrient diffusion, which is desirable in bioactive scaffolds. However, low matrix compaction can compromise the stability of the structures during and after printing using the PAM method, as this process requires a balance between adequate fluidity for extrusion and sufficient rigidity to maintain the deposited shape. In contrast, systems CP8 and pCP8, prepared with higher concentrations of P407 and glycerin, presented a more compact morphology, with thick walls and reduced pore diameter, characteristics that suggest greater structural integrity and dimensional stability after layer deposition.^121–123^ In this context, the pCP8 system stands out, combining the intrinsic porosity of pepsined collagen with the compaction provided by P407 and glycerin, resulting in a ink with the potential to combine good printing fidelity, mechanical support, and favorable biological properties. Thus, while CP8 offers advantages in shape maintenance and controlled release of molecules, pCP2 may be more interesting in applications that require rapid cellular integration but less mechanical stress. Taken together, these results demonstrate that the microstructural architecture observed by SEM is directly related to the expected performance of the formulations in the PAM process, reinforcing the importance of adjusting the composition to balance extrudability, postprinting stability, and biological functionality. In this scenario, the systems proved to be versatile and suitable for application as ink in 3D printing via the PAM method, combining a microstructure that maintains the printed shape well with the ability to support cell adhesion and modulate the release of bioactives.

**Figure 17:**
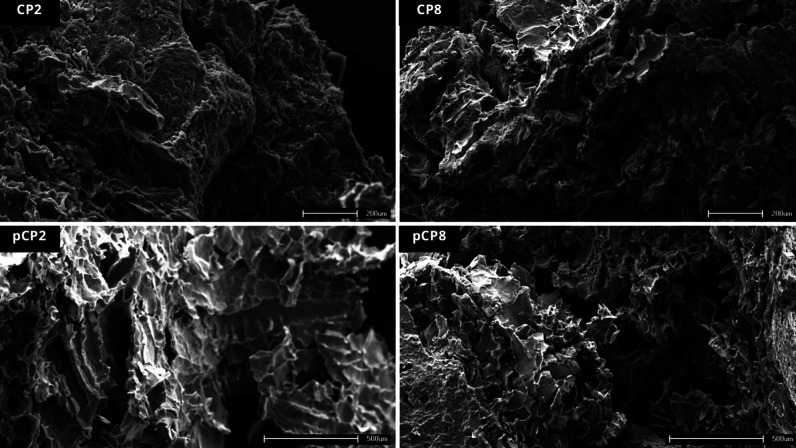
Scanning electron microscopy (SEM) images of ink formulations based on collagen, poloxamer 407, and glycerin, with and without enzymatic extraction by pepsin (CP2, CP8, pCP2 and pCP8).

## Conclusion

All mechanical and rheological properties of systems were dependent on temperature and polymer concentration. Using the Box–Behnken design, it was possible to analyze the effect of each component of the systems (COL or pCOL, P407 and Gly) on the mechanical textural and rheological properties. TPA analysis demonstrated how collagen (COL or pCOL) influenced the hardness, compressibility and adhesiveness values of the systems at low temperatures, while P407 begins to influence them at high temperatures, indicating that as the temperature increases, the collagen decreases the viscosity, and in turn, the micellization process of P407 begins. The synergism between the two polymers is beneficial for the application of the systems in the pharmaceutical and biomedical areas. The rheological parameters analyzed displayed the behavior of each polymeric system when subjected to continuous and oscillatory stress. The temperature, polymer type and concentration of the components in the formulations influenced the rheological properties. The combination of the two polymers resulted in improved systems for pharmaceutical and biomedical applications. For topical application, the CP12 system (0.75/17.5/7%—COL/P407/Gly) presented the best characteristics (i.e., good results in the TPA and rheological profile). This system can come out of the packaging when a force is applied, and the formulation remains in place of administration without flowing at any temperature. The combination of collagen and P407 offers a structural network that can enable the development of innovative systems to be used as drug delivery platforms (e.g., semisolid formulations or pharmaceutical films) or applied on the 3D printing (e.g., CP2, CP8, pCP2 and pCP8). The microstructural characterization of the four best systems demonstrated that adjustments in the composition directly influence the microstructure, mechanical and rheological properties, allowing to balance structural integrity and printing fidelity in inks for 3D printing. In addition to the favorable mechanical and rheological properties demonstrated in this study, the COL–P407 system presents structural and chemical characteristics that make it promising for application as a drug delivery platform. Tilapia collagen, rich in ionizable functional groups, can interact with different classes of bioactive molecules, while P407, due to its amphiphilic nature and micellar behavior, allows the encapsulation of hydrophobic and hydrophilic compounds. This combination gives the system the potential to incorporate and release a wide variety of therapeutic agents, such as small molecules, peptides, proteins and antioxidants, which significantly expands its application possibilities in the pharmaceutical, biomedical and bioprinting areas. Future studies will focus on evaluating the incorporation of therapeutic agents and their effects on the hydrogel’s functional performance, as well as its application in 3D printing of structures for the pharmaceutical and biomedical sectors. Additionally, preliminary biological tests, such as cytotoxicity and skin adhesion tests, will be performed to assess the system’s biocompatibility and safety. These investigations are essential to validate the system’s potential in clinical settings. Therefore, the selected systems represent a promising pipeline for the development of new technologies and innovative treatments in the fields of pharmacy and biomedicine, expanding their applications in various healthcare fields.

## Supplementary Material



## Notes

This document presents the texture profile analysis (TPA) of the Box–Behnken design formulations for COL and pCOL, performed at 10 °C, 25 °C and 34 °C, to evaluate the variations in the textural properties with temperature. Quadratic polynomial equations were used, with significant results when *p* < 0.05. The file includes the values obtained in the TPA analyses. The flow curves of the mono (COL, pCOL, P407) and binary (COL/P407, pCOL/P407) polymer systems are also discussed, providing data on rheological, specific and fluid behavior. These combined analyses provide a detailed view of the physical and rheological properties, fundamental to improving the formulations and ensuring their performance and stability (PDF)

## Abbreviations

COL: collagen acid extraction
pCOL: acid pepsin extraction of collagen
CP: binary polymer system using COL
pCP: binary polymer system using pCOL
