# Supplementary material for: Binary Polymeric System Based on Fish Collagen and Poloxamer 407: Mechanical and Rheological Analysis for Pharmaceutical and Biomedical Applications
Source: ACS Omega. 2025 Nov 5;10(45):53927–58. doi: 10.1021/acsomega.5c04058 (PMC12631358; doi:10.1021/acsomega.5c04058)
Supplement: Supplementary file 1 [file ao5c04058_si_001.pdf]

# Supporting Information

## Binary Polymeric System Based on Fish Collagen and Poloxamer 407: Mechanical and Rheological Analysis for Pharmaceutical and Biomedical Applications

Denise Tiemi Uchida[a], Douglas S. Takano Ogassawara[a], Marcos Luciano Bruschi[a,*]

[a]*Laboratory of Research and Development of Drug Delivery Systems, Department of Pharmacy, State University of Maringa, 87020-900, Maringa, PR, Brazil*

**Corresponding author: Marcos Luciano Bruschi;* mlbruschi@uem.br

### List of Contents

1. Texture profile analysis (TPA) of the Box-Benhken design formulations (both for COL and pCOL) were performed at temperatures of 10, 25 and 34 °C. The quadratic polynomial equations and $R^2$ values are shown in Tables S1-S5 and were considered significant when $p < 0.05$. **(Tables S1-S5)**

2. TPA results of binary polymeric systems composed of tilapia skin collagen (COL or pCOL), poloxamer 407 (P407) and glycerin, at temperatures of 10, 25 and 34 °C. **(Tables S6-S8)**

3. Flow curves of the mono and binary polymeric systems containing collagen obtained by acid extraction (COL), collagen obtained by acid extraction with pepsin (pCOL) and/or poloxamer 407 (P407) at temperatures of 10, 25 and 34 ºC. **(Figure S1-S6)**

**Table S1.** Equation terms and coefficient of determination ($R^2$) of the factorial design for analysis of acid extraction types (with or without pepsin) and hardness (N) values.

| Collagen | T (°C) | Equation | $R^2$ |
|---|---|---|---|
| | | Hardness (N) | |
| COL | 10 | $y = 0.128667 + 0.09800\,X_1 + 0.040542\,X_1^2 + 0.03625\,X_1X_3$ | 0.94221 |
| | 25 | $y = 0.098 + 0.053125\,X_1 + 0.050375\,X_2 + 0.0365\,X_3 + 0.045\,X_2^2 + 0.013\,X_1X_2 + 0.02275\,X_1X_3 + 0.057750\,X_2X_3$ | 0.92104 |
| | 34 | $y = 0.046 + 0.0035\,X_1 + 0.14775\,X_2 + 0.0160\,X_3 - 0.0100\,X_1^2 + 0.1455\,X_2^2 + 0.01\,X_3^2 + 0.0085\,X_1X_2 - 0.003\,X_1X_3 + 0.027\,X_2X_3$ | 0.98898 |
| pCOL | 10 | $y = 0.052667 + 0.0055\,X_1 - 0.003208\,X_3^2$ | 0.9053 |
| | 25 | $y = 0.052667 + 0.02900\,X_2 + 0.026625\,X_3 - 0.024458\,X_1^2 + 0.027792\,X_2^2 + 0.025542\,X_3^2 + 0.0525\,X_2X_3$ | 0.73662 |
| | 34 | $y = 0.045667 - 0.00825\,X_1 + 0.154625\,X_2 + 0.005125X_3 + 0.153417\,X_2^2 + 0.005417\,X_3^2 - 0.01325\,X_1X_2 +$ | 0.99801 |

Note: COL: Collagen Acid Extraction; pCOL: Acid Pepsin Extraction of Collagen; T: Temperature.

**Table S2.** Equation terms and coefficient of determination ($R^2$) of the factorial design for analysis of acid extraction types (with or without pepsin) and compressibility (N.mm) values.

| Collagen | T (°C) | Equation | $R^2$ |
|---|---|---|---|
| | | Compressibility (N.mm) | |
| COL | 10 | $y = 0.34633 + 0.329875\,X_1$ | 0.93023 |
| | 25 | $y = 0.261 + 0.174\,X_1 + 0.222375\,X_2 - 0.04775\,X_1^2 + 0.199\,X_2^2 + 0.07225\,X_1X_3 + 0.2830\,X_2X_3$ | 0.8789 |
| | 34 | $y = 0.050333 + 0.017125\,X_1 + 0.6725\,X_2 + 0.056375\,X_3 - 0.030542\,X_1^2 + 0.673708\,X_2^2 + 0.042958\,X_3^2 + 0.0305\,X_1X_2 + 0.0845\,X_2X_3$ | 0.99382 |
| pCOL | 10 | $y = 0.0970 + 0.0210\,X_1$ | 0.87913 |
| | 25 | $y = 0.091667 + 0.012875\,X_1 + 0.142125\,X_2 + 0.128500\,X_3 - 0.123583\,X_1^2 + 0.134917\,X_2^2 + 0.134667\,X_3^2 + 0.25625\,X_2X_3$ | 0.74174 |
| | 34 | $y = 0.069 - 0.02825\,X_1 + 0.7565\,X_2 - 0.046125\,X_1^2 + 0.746875\,X_2^2 + 0.051875\,X_3^2 - 0.04525\,X_1X_2$ | 0.99643 |

Note: COL: Collagen Acid Extraction; pCOL: Acid Pepsin Extraction of Collagen; T: Temperature.

**Table S3.** Equation terms and coefficient of determination ($R^2$) of the factorial design for analysis of acid extraction types (with or without pepsin) and adhesiveness values (N.mm).

| Collagen | T (°C) | Equation | $R^2$ |
|---|---|---|---|
| | | Adhesiveness (N.mm) | |
| COL | 10 | $y = 0.0980 + 0.1105\, X_1 + 0.037875\, X_1^2 + 0.03225\, X_1X_3$ | 0.91652 |
| | 25 | $y = 0.087667 + 0.081\, X_1 + 0.152125\, X_2 + 0.135125\, X_3 - 0.077208\, X_1^2 + 0.146042\, X_2^2 + 0.079042\, X_3^2 + 0.24425\, X_2X_3$ | 0.80782 |
| | 34 | $y = 0.633625\, X_2 + 0.633625\, X_2^2$ | 0.99328 |
| pCOL | 10 | $y = 0.0100\, X_1 + 0.0100\, X_1^2$ | 0.93973 |
| | 25 | $y = 0.000$ | 0.74898 |
| | 34 | $y = 0.736875\, X_2 + 0.736875\, X_2^2$ | 0.99655 |

Note: COL: Collagen Acid Extraction; pCOL: Acid Pepsin Extraction of Collagen; T: Temperature.

**Table S4.** Equation terms and coefficient of determination ($R^2$) of the factorial design for analysis of acid extraction types (with or without pepsin) and elasticity (mm) values.

| Collagen | T (°C) | Equation | $R^2$ |
|---|---|---|---|
| | | Elasticity (mm) | |
| COL | 10 | $y = 1.345667$ | 0.72908 |
| | 25 | $y = 1.16733 - 0.027625\, X_1 + 0.072125\, X_2 + 0.0140\, X_3 + 0.080208\, X_1^2 + 0.077708\, X_2^2 - 0.089042\, X_3^2 - 0.109250\, X_1X_2 + 0.0125\, X_1X_3$ | 0.76851 |
| | 34 | $y = 1.245333$ | 0.73981 |
| pCOL | 10 | $y = 1.183667 - 0.122500\, X_1$ | 0.8378 |
| | 25 | $y = 1.273667$ | 0.45349 |
| | 34 | $y = 1.06833$ | 0.76936 |

Note: COL: Collagen Acid Extraction; pCOL: Acid Pepsin Extraction of Collagen; T: Temperature.

**Table S5.** Terms of the equation and coefficient of determination ($R^2$) of the factorial design for analysis of the types of acid extraction (with or without pepsin) and cohesiveness values (dimensionless).

| Collagen | T (°C) | Equation | $R^2$ |
|---|---|---|---|
| | | Cohesiveness (dimensionless) | |
| COL | 10 | $y = 0.983667$ | 0.85309 |
| | 25 | $y = 1.017333$ | 0.75189 |
| | 34 | $y = 1.044333$ | 0.68294 |
| pCOL | 10 | $y = 1.036333 + 0.0.038750\ X_2 - 0.03975\ X_3 - 0.06125\ X_2X_3$ | 0.84177 |
| | 25 | $y = 1.017 + 0.0275\ X_1$ | 0.62389 |
| | 34 | $y = 0.9800$ | 0.71495 |

Note: COL: Collagen Acid Extraction; pCOL: Acid Pepsin Extraction of Collagen; T: Temperature.

**Table S6.** Texture profile analysis (TPA) of binary polymeric systems composed of tilapia skin collagen (COL or pCOL), poloxamer 407 (P407) and glycerin, at temperatures (T) of 10 °C.

| CP or pCP | Collagen (%,w/w) | P407 (%,w/w) | Glycerin (%,w/w) | Hardness (N) | | Compressibility (N.mm) | | Adhesiveness (N.mm) | | Cohesiveness (dimensionless) | | Elasticity (mm) | |
|---|---|---|---|---|---|---|---|---|---|---|---|---|---|
| | | | | COL | pCOL | COL | pCOL | COL | pCOL | COL | pCOL | COL | pCOL |
| 1 | 0.5 | 12.5 | 5 | 0.071±0.004 | 0.048±0.003 | 0.158±0.007 | 0.079±0.006 | 0.009±0.001 | 0.0±0.0 | 1.203±0.034 | 1.308±0.072 | 1.025±0.032 | 0.859±0.056 |
| 2 | 1 | 12.5 | 5 | 0.269±0.017 | 0.058±0.008 | 0.834±0.046 | 0.116±0.014 | 0.241±0.008 | 0.006±0.002 | 1.467±0.047 | 1.169±0.044 | 1.002±0.008 | 1.002±0.049 |
| 3 | 0.5 | 17.5 | 5 | 0.054±0.001 | 0.046±0.002 | 0.097±0.006 | 0.076±0.004 | 0.010±0.001 | 0.0±0.0 | 1.203±0.062 | 1.368±0.122 | 1.008±0.017 | 1.021±0.054 |
| 4 | 1 | 17.5 | 5 | 0.345±0.005 | 0.062±0.004 | 1.117±0.035 | 0.133±0.004 | 0.353±0.023 | 0.025±0.002 | 1.465±0.097 | 1.189±0.085 | 0.996±0.002 | 1.057±0.023 |
| 5 | 0.5 | 15 | 3 | 0.074±0.002 | 0.048±0.002 | 0.160±0.012 | 0.074±0.004 | 0.029±0.003 | 0.0±0.0 | 1.294±0.019 | 1.395±0.143 | 0.994±0.007 | 1.07±0.037 |
| 6 | 1 | 15 | 3 | 0.149±0.011 | 0.055±0.002 | 0.403±0.017 | 0.115±0.012 | 0.119±0.008 | 0.023±0.002 | 1.328±0.085 | 1.227±0.091 | 1.019±0.026 | 1.066±0.051 |
| 7 | 0.5 | 15 | 7 | 0.050±0.003 | 0.044±0.001 | 0.082±0.019 | 0.071±0.002 | 0.031±0.001 | 0.0±0.0 | 1.194±0.126 | 1.717±0.193 | 1.054±0.181 | 0.984±0.048 |
| 8 | 1 | 15 | 7 | 0.270±0.015 | 0.055±0.004 | 0.782±0.055 | 0.104±0.003 | 0.250±0.018 | 0.026±0.003 | 1.413±0.138 | 1.223±0.042 | 1.006±0.002 | 0.947±0.097 |
| 9 | 0.75 | 12.5 | 3 | 0.100±0.007 | 0.047±0.001 | 0.252±0.018 | 0.087±0.01 | 0.077±0.008 | 0.0±.0.0 | 1.256±0.066 | 1.266±0.107 | 0.989±0.057 | 0.98±0.039 |
| 10 | 0.75 | 17.5 | 3 | 0.114±0.003 | 0.047±0.001 | 0.279±0.016 | 0.098±0.003 | 0.074±0.005 | 0.0±0.0 | 1.264±0.033 | 1.218±0.101 | 1.013±0.005 | 1.149±0.086 |
| 11 | 0.75 | 12.5 | 7 | 0.095±0.005 | 0.049±0.004 | 0.245±0.018 | 0.104±0.009 | 0.081±0.006 | 0.0±0.0 | 1.195±0.075 | 1.208±0.085 | 1.010±0.034 | 1.046±0.037 |
| 12 | 0.75 | 17.5 | 7 | 0.134±0.002 | 0.054±0.004 | 0.360±0.029 | 0.123±0.014 | 0.115±0.011 | 0.0±0.0 | 1.345±0.083 | 1.2±0.108 | 0.994±0.032 | 0.97±0.051 |
| 13 | 0.75 | 15 | 5 | 0.113±0.005 | 0.052±0.001 | 0.275±0.013 | 0.088±0.004 | 0.087±0.007 | 0.0±0.0 | 1.260±0.098 | 1.268±0.117 | 0.968±0.039 | 1.015±0.016 |
| 14 | 0.75 | 15 | 5 | 0.127±0.002 | 0.052±0.002 | 0.369±0.022 | 0.101±0.0 | 0.115±0.006 | 0.0±0.0 | 1.299±0.033 | 1.132±0.049 | 0.987±0.013 | 1.052±0.072 |
| 15 | 0.75 | 15 | 5 | 0.146±0.012 | 0.054±0.002 | 0.395±0.031 | 0.102±0.007 | 0.092±0.003 | 0.0±0.0 | 1.478±0.113 | 1.151±0.111 | 0.996±0.006 | 1.042±0.089 |

Note: CP, Binary polymer system using COL; pCP, Binary polymer system using pCOL; COL: Collagen Acid Extraction; pCOL: Acid Pepsin Extraction of Collagen; P407: Poloxamer 407; T: Temperature.

**Table S7.** Texture profile analysis (TPA) of binary polymeric systems composed of tilapia skin collagen (COL or pCOL), poloxamer 407 (P407) and glycerin, at temperatures (T) of 25 ºC.

| CP or pCP | Collagen (%, w/w) | P407 (%,w/w) | Glycerin (%,w/w) | Hardness (N) | | Compressibility (N.mm) | | Adhesiveness (N.mm) | | Cohesiveness (dimensionless) | | Elasticity (mm) | |
|---|---|---|---|---|---|---|---|---|---|---|---|---|---|
| | | | | COL | pCOL | COL | pCOL | COL | pCOL | COL | pCOL | COL | pCOL |
| 1 | 0.5 | 12.5 | 5 | 0.060±0.002 | 0.052±0.004 | 0.116±0.003 | 0.079±0.006 | 0.020±0.001 | 0.0±0.0 | 1.157±0.029 | 1.248±0.075 | 0.996±0.039 | 0.993±0.083 |
| 2 | 1 | 12.5 | 5 | 0.165±0.004 | 0.053±0.008 | 0.477±0.032 | 0.094±0.008 | 0.189±0.006 | 0.0±0.0 | 1.220±0.033 | 1.156±0.024 | 1.027±0.050 | 1.051±0.036 |
| 3 | 0.5 | 17.5 | 5 | 0.097±0.006 | 0.056±0.001 | 0.304±0.033 | 0.105±0.006 | 0.111±0.007 | 0.0±0.0 | 1.649±0.094 | 1.282±0.033 | 1.033±0.046 | 1.014±0.104 |
| 4 | 1 | 17.5 | 5 | 0.254±0.018 | 0.063±0.001 | 0.752±0.036 | 0.134±0.003 | 0.306±0.011 | 0.049±0.007 | 1.275±0.058 | 1.277±0.017 | 0.990±0.022 | 1.033±0.092 |
| 5 | 0.5 | 15 | 3 | 0.063±0.005 | 0.050±0.006 | 0.113±0.009 | 0.088±0.002 | 0.027±0.003 | 0.0±0.0 | 1.138±0.025 | 1.102±0.064 | 1.004±0.079 | 0.981±0.07 |
| 6 | 1 | 15 | 3 | 0.099±0.004 | 0.059±0.001 | 0.262±0.013 | 0.123±0.013 | 0.103±0.005 | 0.0±0.0 | 1.158±0.028 | 1.113±0.113 | 0.988±0.040 | 1.031±0.121 |
| 7 | 0.5 | 15 | 7 | 0.055±0.005 | 0.048±0.004 | 0.094±0.018 | 0.088±0.007 | 0.010±0.005 | 0.0±0.0 | 1.134±0.094 | 1.204±0.052 | 0.952±0.046 | 0.926±0.097 |
| 8 | 1 | 15 | 7 | 0.182±0.020 | 0.058±0.003 | 0.532±0.039 | 0.112±0.005 | 0.218±0.007 | 0.012±0.001 | 1.204±0.024 | 1.278±0.055 | 1.002±0.003 | 1.019±0.041 |
| 9 | 0.75 | 12.5 | 3 | 0.078±0.004 | 0.050±0.003 | 0.174±0.017 | 0.09±0.009 | 0.059±0.003 | 0.0±0.0 | 1.128±0.066 | 1.194±0.018 | 1.036±0.057 | 0.989±0.044 |
| 10 | 0.75 | 17.5 | 3 | 0.101±0.003 | 0.054±0.003 | 0.266±0.010 | 0.113±0.010 | 0.075±0.007 | 0.0±0.0 | 1.149±0.034 | 1.209±0.020 | 1.016±0.054 | 0.964±0.093 |
| 11 | 0.75 | 12.5 | 7 | 0.071±0.003 | 0.053±0.002 | 0.162±0.007 | 0.097±0.006 | 0.062±0.004 | 0.0±0.0 | 1.169±0.024 | 1.220±0.077 | 0.964±0.049 | 1.126±0.152 |
| 12 | 0.75 | 17.5 | 7 | 0.325±0.024 | 0.267±0.016 | 1.386±0.108 | 1.145±0.13 | 1.055±0.088 | 0.847±0.042 | 1.178±0.092 | 1.235±0.096 | 1.018±0.029 | 0.975±0.019 |
| 13 | 0.75 | 15 | 5 | 0.095±0.003 | 0.053±0.004 | 0.240±0.014 | 0.091±0.007 | 0.071±0.001 | 0.0±0.0 | 1.169±0.022 | 1.249±0.103 | 0.995±0.065 | 1.032±0.035 |
| 14 | 0.75 | 15 | 5 | 0.101±0.004 | 0.050±0.005 | 0.279±0.013 | 0.094±0.01 | 0.109±0.006 | 0.0±.0.0 | 1.164±0.039 | 1.416±0.026 | 1.004±0.019 | 1.0±0.072 |
| 15 | 0.75 | 15 | 5 | 0.098±0.001 | 0.055±0.004 | 0.264±0.010 | 0.09±0.005 | 0.083±0.004 | 0.0±0.0 | 1.169±0.030 | 1.156±0.094 | 1.053±0.035 | 1.019±0.019 |

Note: CP, Binary polymer system using COL; pCP, Binary polymer system using pCOL; COL: Collagen Acid Extraction; pCOL: Acid Pepsin Extraction of Collagen; P407: Poloxamer 407; T: Temperature.

**Table S8.** Texture profile analysis (TPA) of binary polymeric systems composed of tilapia skin collagen (COL or pCOL), poloxamer 407 (P407) and glycerin, at temperatures (T) of 34 ºC.

| CP or pCP | Collagen (%, w/w) | P407 (%,w/w) | Glycerin (%,w/w) | Hardness (N) | | Compressibility (N.mm) | | Adhesiveness (N.mm) | | Cohesiveness (dimensionless) | | Elasticity (mm) | |
|---|---|---|---|---|---|---|---|---|---|---|---|---|---|
| | | | | COL | pCOL | COL | pCOL | COL | pCOL | COL | pCOL | COL | pCOL |
| 1 | 0.5 | 12.5 | 5 | 0.045±0.001 | 0.046±0.003 | 0.053±0.001 | 0.07±0.007 | 0.000±0.000 | 0.0±0.0 | 1.116±0.066 | 1.085±0.049 | 0.934±0.082 | 0.987±0.024 |
| 2 | 1 | 12.5 | 5 | 0.045±0.001 | 0.043±0.003 | 0.064±0.002 | 0.052±0.001 | 0.000±0.000 | 0.0±0.0 | 1.011±0.033 | 1.055±0.097 | 0.975±0.091 | 1.002±0.037 |
| 3 | 0.5 | 17.5 | 5 | 0.301±0.024 | 0.374±0.014 | 1.262±0.085 | 1.578±0.104 | 1.144±0.076 | 1.457±0.02 | 1.046±0.030 | 1.003±0.014 | 0.992±0.016 | 0.988±0.008 |
| 4 | 1 | 17.5 | 5 | 0.335±0.021 | 0.318±0.017 | 1.395±0.073 | 1.379±0.028 | 1.251±0.091 | 1.302±0.052 | 1.052±0.053 | 1.012±0.039 | 1.021±0.000 | 1.015±0.012 |
| 5 | 0.5 | 15 | 3 | 0.043±0.001 | 0.047±0.002 | 0.055±0.006 | 0.074±0.002 | 0.000±0.000 | 0.0±0.0 | 1.561±0.026 | 1.026±0.085 | 0.992±0.011 | 1.027±0.049 |
| 6 | 1 | 15 | 3 | 0.046±0.003 | 0.044±0.001 | 0.056±0.006 | 0.062±0.002 | 0.000±0.000 | 0.0±0.0 | 1.176±0.083 | 1.039±0.035 | 1.076±0.079 | 0.996±0.033 |
| 7 | 0.5 | 15 | 7 | 0.052±0.005 | 0.051±0.006 | 0.074±0.001 | 0.08±0.006 | 0.000±0.000 | 0.0±0.0 | 1.246±0.091 | 1.178±0.083 | 1.012±0.009 | 1.060±0.1 |
| 8 | 1 | 15 | 7 | 0.043±0.001 | 0.047±0.001 | 0.066±0.005 | 0.083±0.009 | 0.000±0.000 | 0.0±.0 | 1.087±0.038 | 1.318±0.088 | 1.001±0.111 | 1.020±0.08 |
| 9 | 0.75 | 12.5 | 3 | 0.039±0.002 | 0.048±0.004 | 0.036±0.003 | 0.069±0.001 | 0.000±0.000 | 0.0±0.0 | 1.127±0.142 | 1.050±0.081 | 1.048±0.059 | 0.951±0.057 |
| 10 | 0.75 | 17.5 | 3 | 0.303±0.016 | 0.344±0.047 | 1.287±0.036 | 1.674±0.190 | 1.131±0.035 | 1.515±0.159 | 1.069±0.029 | 1.058±0.002 | 0.999±0.025 | 1.043±0.031 |
| 11 | 0.75 | 12.5 | 7 | 0.046±0.003 | 0.044±0.001 | 0.078±0.001 | 0.058±0.004 | 0.000±0.000 | 0.0±0.0 | 1.136±0.008 | 1.194±0.057 | 0.942±0.034 | 1.025±0.057 |
| 12 | 0.75 | 17.5 | 7 | 0.418±0.034 | 0.382±0.009 | 1.667±0.078 | 1.670±0.08 | 1.543±0.133 | 1.621±0.096 | 1.085±0.022 | 0.99±0.024 | 1.005±0.014 | 0.99±0.012 |
| 13 | 0.75 | 15 | 5 | 0.046±0.003 | 0.047±0.002 | 0.045±0.004 | 0.073±0.001 | 0.000±0.000 | 0.0±0.0 | 1.211±0.121 | 1.012±0.1 | 0.972±0.108 | 0.998±0.027 |
| 14 | 0.75 | 15 | 5 | 0.047±0.001 | 0.043±0.001 | 0.054±0.005 | 0.06±0.007 | 0.000±0.000 | 0.0±0.0 | 1.153±0.141 | 1.112±0.14 | 1.065±0.057 | 0.996±0.102 |
| 15 | 0.75 | 15 | 5 | 0.045±0.003 | 0.047±0.001 | 0.052±0.004 | 0.074±0.008 | 0.000±0.000 | 0.0±0.0 | 1.372±0.094 | 1.081±0.071 | 1.096±0.066 | 0.946±0.102 |

Note: CP, Binary polymer system using COL; pCP, Binary polymer system using pCOL; COL: Collagen Acid Extraction; pCOL: Acid Pepsin Extraction of Collagen; P407: Poloxamer 407; T: Temperature.

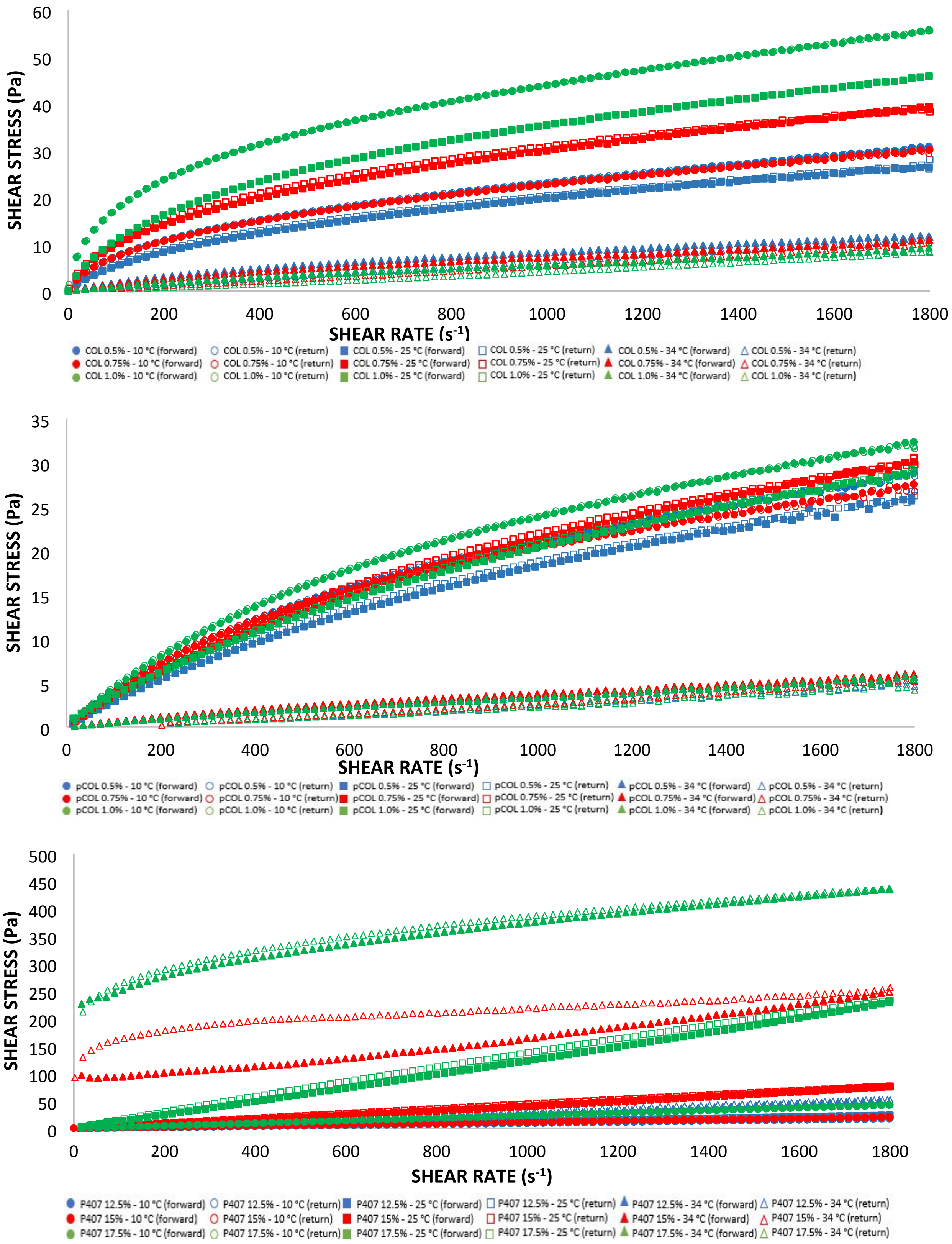


**Figure S1.** Flow curves of the monopolymeric systems collagen obtained by acid extraction (COL), collagen obtained by acid extraction with pepsin (pCOL) and poloxamer 407 (P407) at temperatures of 10, 25 and 34 ºC. The closed symbol represents the forward curve, and the open symbol represents the return curve. Each flow curve is the average of at least three replicates.

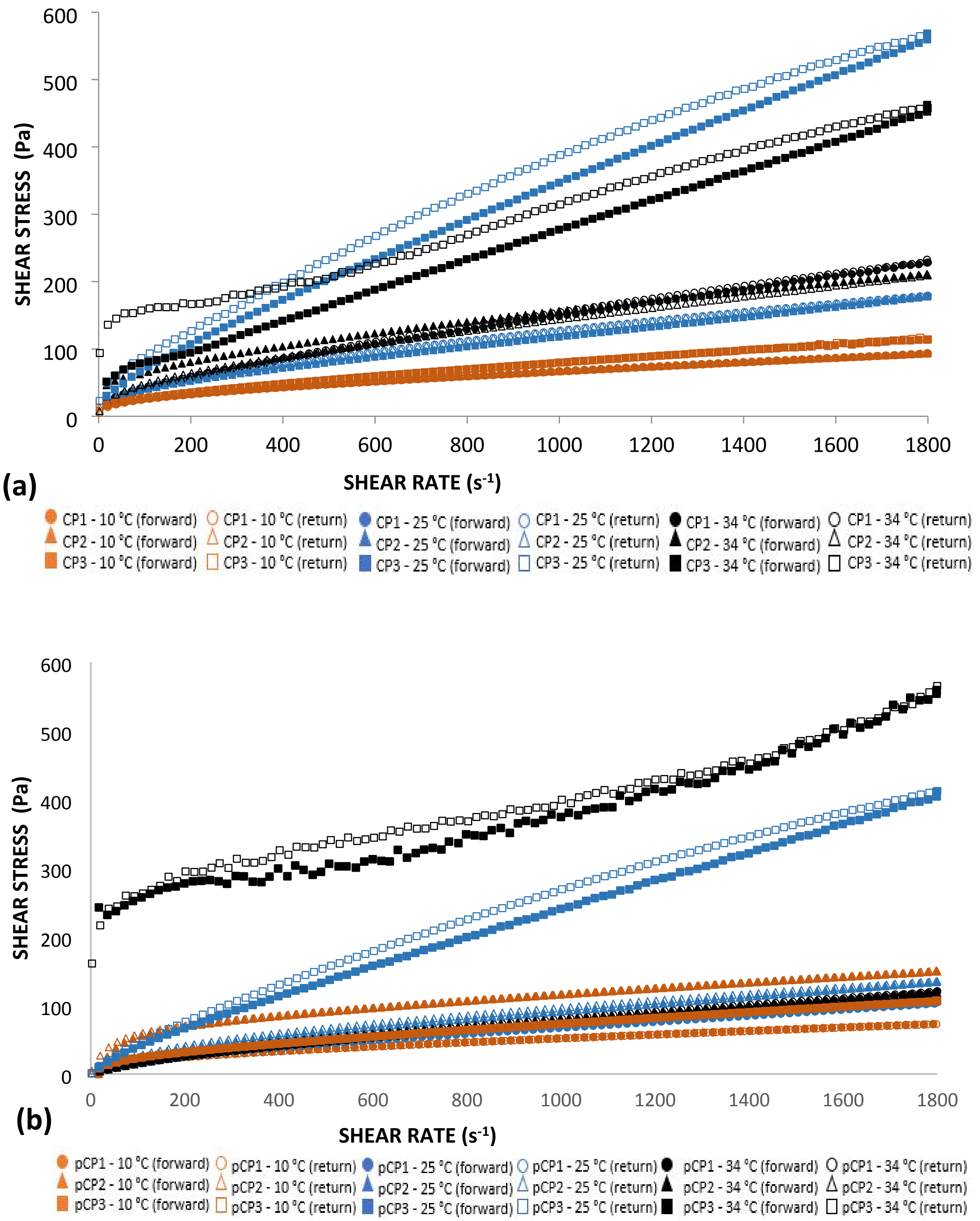


**Figure S2.** Flow curves of binary systems with collagen obtained by acid and acid extraction with pepsin at temperatures of 10, 25 and 34 ºC: (a) CP1 – CP3; (b) pCP1 – pCP3. The closed symbol represents the outward curve, and the open symbol represents the return curve. Each flow curve is the average of at least three replicates.

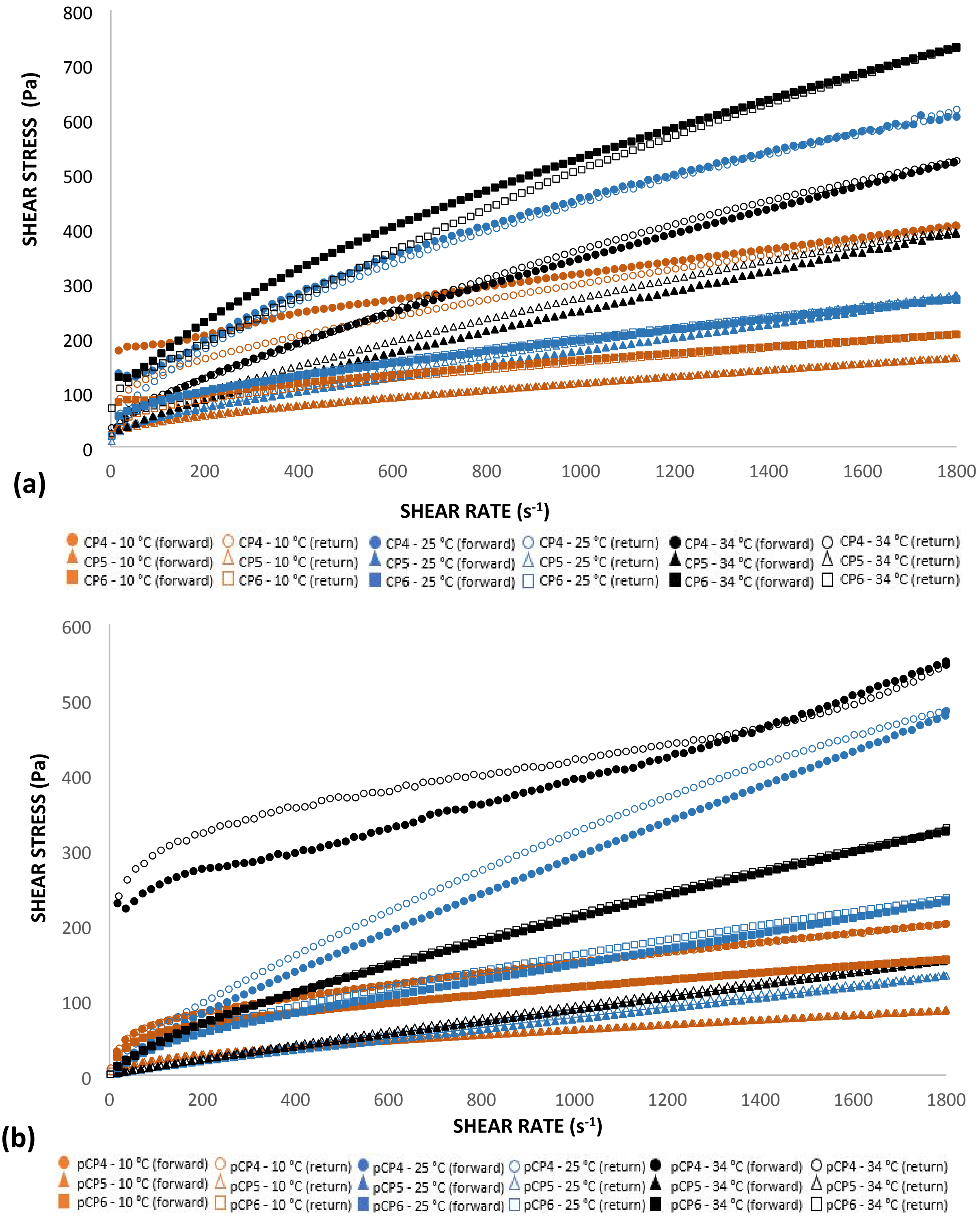


**Figure S3.** Flow curves of binary systems with collagen obtained from acid and acid extraction with pepsin at temperatures of 10, 25 and 34 ºC: (a) CP4 – CP6; (b) pCP4 – pCP6. The closed symbol represents the outward curve, and the open symbol represents the return curve. Each flow curve is the average of at least three replicates.

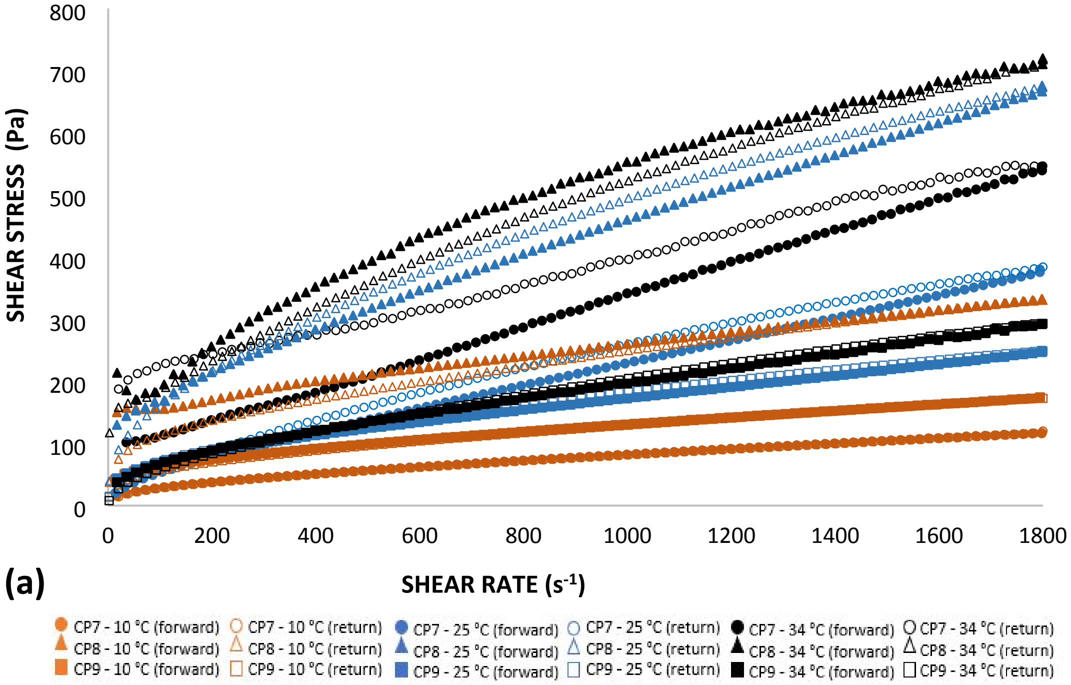


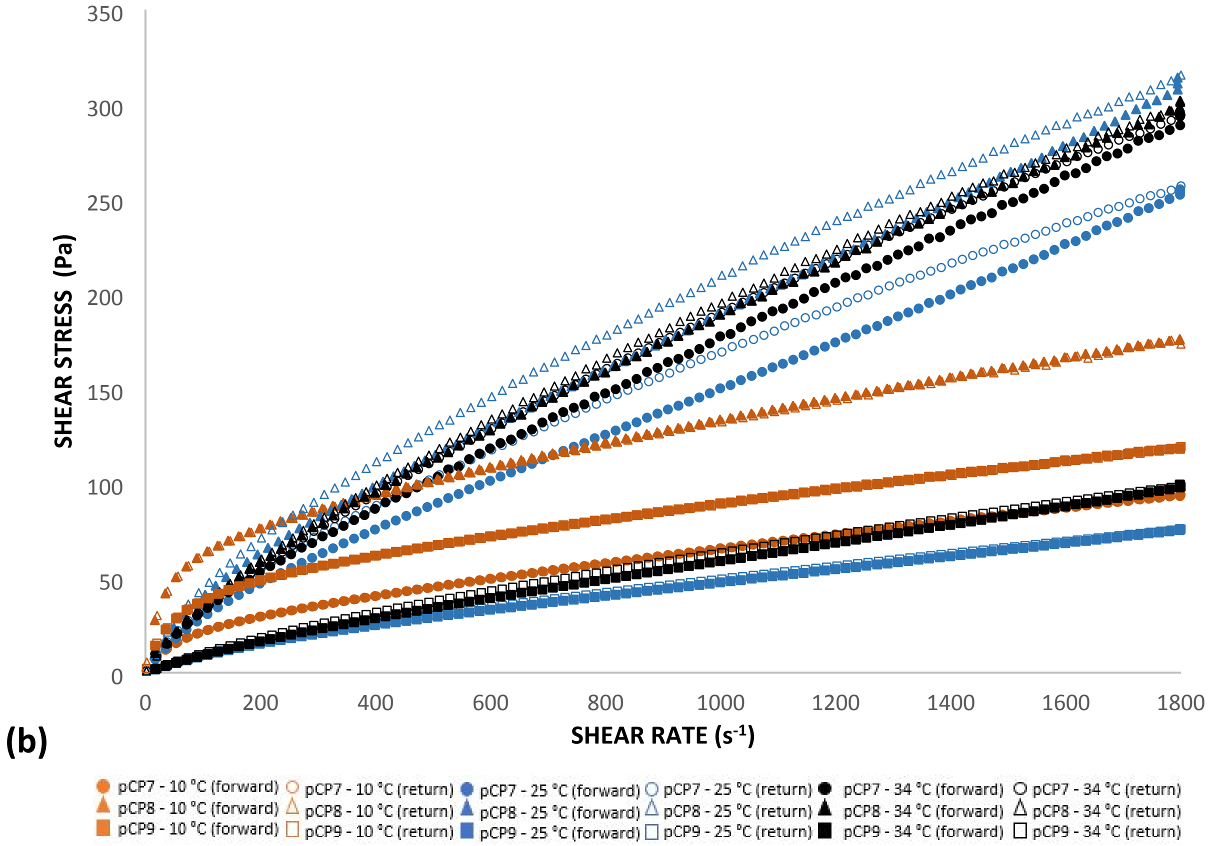


**Figure S4.** Flow curves binary systems with collagen obtained from acid and acid extraction with pepsin at temperatures of 10, 25 and 34 ºC: (a) CP7 – CP9; (b) pCP7 – pCP9. The closed symbol represents the outward curve, and the open symbol represents the return curve. Each flow curve is the average of at least three replicates.

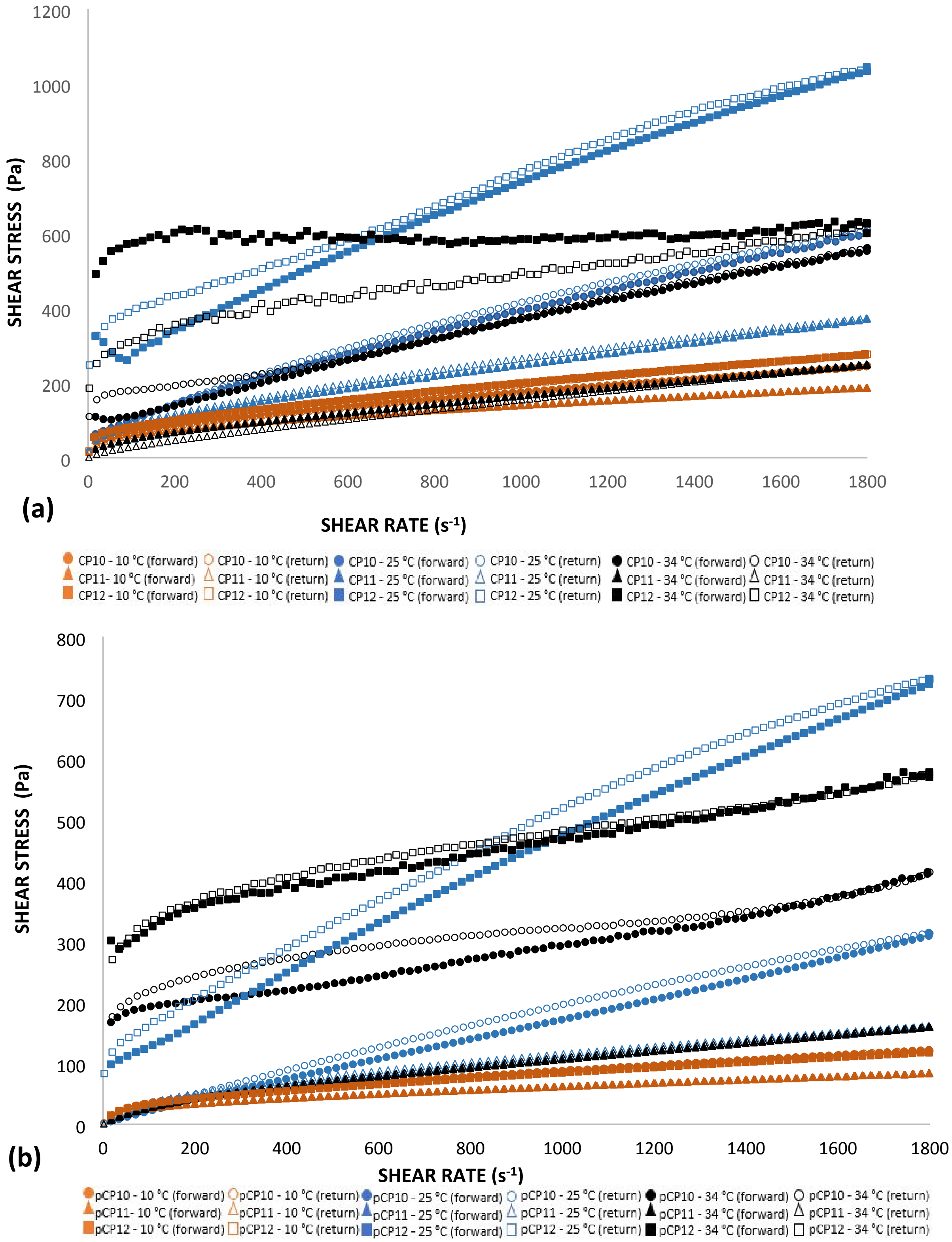


**Figure S5.** Flow curves of binary systems with collagen obtained from acid and acid extraction with pepsin at temperatures of 10, 25 and 34 ºC; (a) CP10 – CP12; (b) pCP10 – pCP12. The closed symbol represents the outward curve, and the open symbol represents the return curve. Each flow curve is the average of at least three replicates.

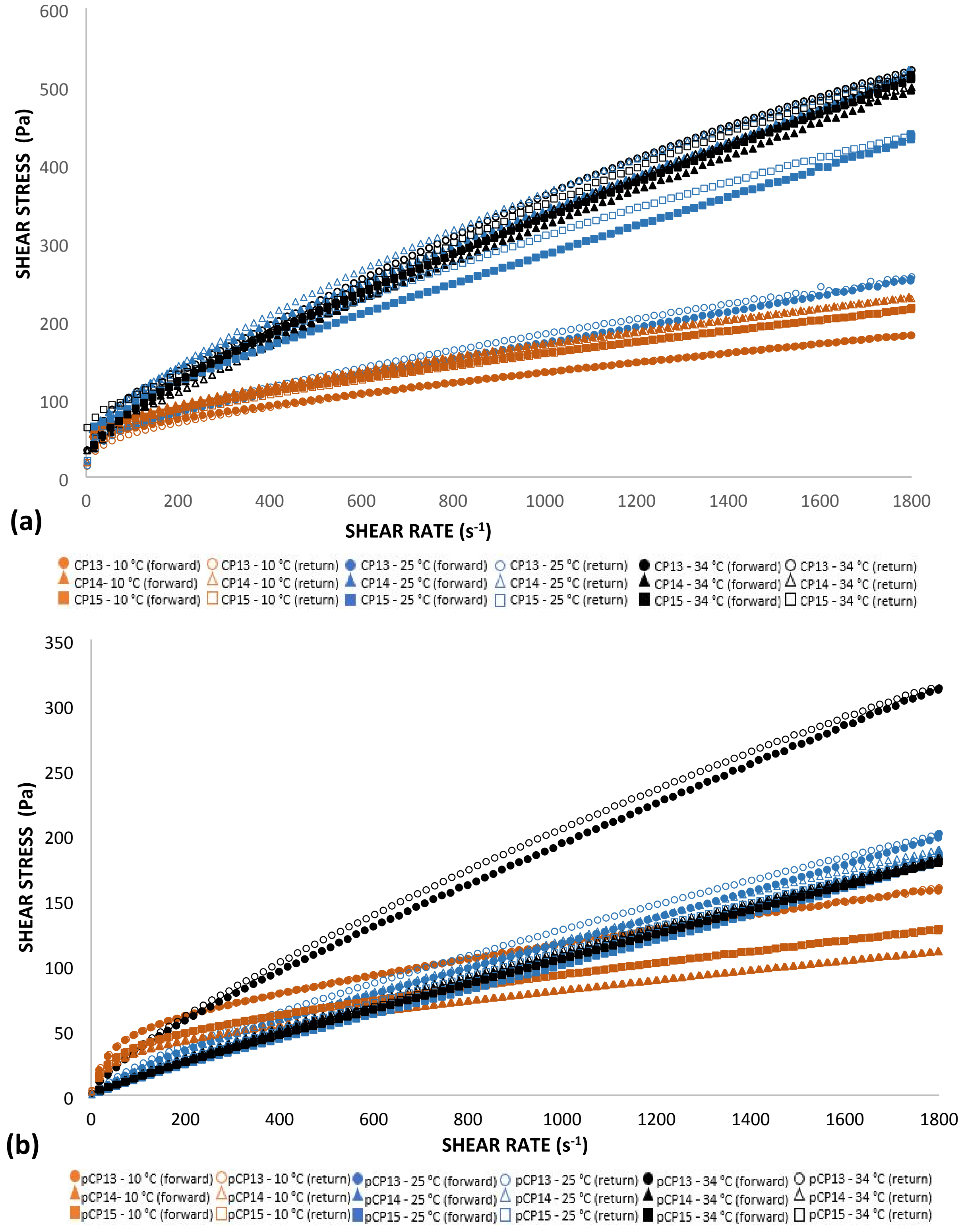


**Figure S6.** Flow curves of binary systems with collagen obtained from acid and acid extraction with pepsin at temperatures of 10, 25 and 34 ºC: (a) CP13 – CP15; (b) pCP13 – pCP15. The closed symbol represents the outward curve, and the open symbol represents the return curve. Each flow curve is the average of at least three replicates.
